# Improving learning outcomes of medical terminology course through classroom-based gamified crossword puzzle activities

**DOI:** 10.3389/fmed.2026.1705623

**Published:** 2026-01-16

**Authors:** Aziz Jamal, Ning Liu, Yunfei Li

**Affiliations:** 1 Health Administration Program, Faculty of Business & Management, Universiti Teknologi MARA Puncak Alam Campus, Selangor, Malaysia; 2 Department of Environmental Epidemiology, University of Occupational and Environmental Health, Fukuoka, Japan; 3 Department of Neurobiology, Care Sciences and Society, Karolinska Institutet, Stockholm, Sweden

**Keywords:** crossword puzzles, health administration, learning aids, medical terminology, student performance, treatment effect

## Abstract

**Introduction:**

Active learning strategies are widely promoted to enhance student engagement and knowledge retention in higher education. In health administration education, mastery of medical terminology is essential, yet students often experience difficulty with recall and application. Crossword puzzles have been proposed as a practical instructional tool to support terminology learning, but empirical evidence in this context remains limited.

**Methods:**

This study examined the association between the use of crossword puzzles as learning aids and academic performance in a medical terminology course. A non-equivalent control group post-test-only design was employed. The sample comprised 211 s-year undergraduate health administration students enrolled in a 14-week course. Paper-based crossword puzzles were introduced in week 5 and implemented over 9 weeks. Independent t-tests were used to examine group differences, followed by multiple linear regression analysis to adjust for relevant covariates. Treatment effect analyses estimated the average treatment effect (ATE), average treatment effect on the treated (ATET), and average treatment effect on the non-treated (ATENT).

**Result:**

Students who used crossword puzzles demonstrated higher scores across individual assessment components and total test scores compared with the control group (*p* < 0.001). Regression-adjusted analyses confirmed statistically significant differences in total test scores between groups (*p* < 0.001). Treatment effect analyses yielded predominantly positive, statistically significant estimates for total scores, including ATE, ATET, and ATENT (*p* < 0.01), indicating consistent associations between crossword use and test performance.

**Discussion:**

The findings indicate that crossword puzzles are positively associated with higher performance in medical terminology assessments, supporting their role as a supplementary learning strategy. Students with weaker foundational knowledge may require additional instructional support. Given the observational study design, the findings should be interpreted as associative rather than causal.

## 1 Introduction

Crossword puzzles have long been recognized as an engaging and interactive educational tool that enhances learning across various disciplines. Their integration into academic settings has been supported by research demonstrating improvements in knowledge retention, student motivation, and overall satisfaction. As a low-cost and enjoyable teaching aid, crosswords provide an alternative or complementary instructional approach, particularly in fields that require mastery of technical vocabulary and conceptual understanding. Studies have shown that crossword puzzles contribute significantly to knowledge retention. For example, Zamani et al. (1) reported that speech therapy students who used crosswords alongside traditional lectures demonstrated higher immediate and long-term knowledge scores than those who received standard instruction alone. Similarly, Khaewratana (2) found that crosswords facilitate learning by reducing cognitive load, allowing students to concentrate on higher-order concepts rather than struggling with terminology.

In addition to retention, crossword puzzles enhance student engagement. Their gamified nature makes learning less intimidating and more interactive, fostering motivation and sustained participation (3). In computer programming education, crosswords have been shown to accelerate learning and maintain student enthusiasm throughout an academic year (4). Given their adaptability, crosswords have been successfully employed in diverse disciplines, including medical education, where they serve as both a supplementary learning tool and a formative assessment method (5). Crosswords have been widely used across different educational levels, from early education to higher education. In primary education, they help students practice spelling, vocabulary acquisition, and comprehension while fostering a sense of accomplishment (6–8). With advancements in technology, interactive puzzles supported by Web 2.0 tools have further increased student engagement and accessibility (9).

At the university level, crosswords have been incorporated into coursework to reinforce learning, improve retention, and enhance student satisfaction. For instance, in speech therapy and computer engineering courses, students using crosswords have demonstrated superior learning outcomes compared to traditional teaching methods (1, 4). Despite their benefits, some educators face challenges in designing effective crossword-based activities, particularly when integrating new technologies (9). In medical and health-related education, crossword puzzles serve as an engaging strategy to reinforce lecture content and improve comprehension. Several studies highlight their effectiveness in increasing student knowledge, engagement, and exam performance. For instance, Sannathimmappa (10) reported that 86% of microbiology and immunology students perceived an improvement in their examination grades due to crossword puzzle activities. Similarly, during the COVID-19 pandemic, crosswords were used as an innovative online learning tool, successfully maintaining student interest in physiology courses (11).

Crosswords also function as a formative assessment tool, allowing students to self-evaluate their comprehension and establish connections between concepts. In a nursing program, a crossword puzzle tournament was employed as an exam preparation strategy, improving students’ understanding of key concepts and their ability to prioritize patient care (12). While some students may find the format initially challenging, proper guidance and practice can maximize its educational benefits (13). Crossword puzzles contribute to improved memory retention by engaging cognitive processes that enhance learning and recall. Solving crosswords requires active retrieval of information, which strengthens neural connections and supports long-term retention (14). Moreover, crosswords facilitate vocabulary retention by emphasizing word meanings and spellings, making them particularly effective for learning technical terms in science, technology, engineering, and mathematics (STEM) fields (15).

Studies have also demonstrated that students who regularly create and solve crosswords exhibit improved quiz scores and achieve high learning objectives (16). Furthermore, compared to some computerized brain-training games, crossword puzzles have been found to be more effective in enhancing cognitive function in older adults, indicating their potential benefits across different age groups (17). However, while crosswords alone have demonstrated strong educational value, additional elaboration techniques do not consistently enhance retention, suggesting that their fundamental structure is already an effective learning mechanism (2). Despite the demonstrated benefits of crossword puzzles in education, a recent systematic review by Arnold et al. (18) identified a critical gap in the existing research. Many studies have primarily assessed outcomes based on student perceptions and self-assessed improvements rather than objective measures of learning. While self-reported confidence and competence may show substantial differences in response to educational interventions, they are weak surrogates for actual academic achievement. Furthermore, non-randomized studies that used test scores to evaluate the effectiveness of crosswords often suffered from methodological issues. Specifically, most studies failed to account for subject covariates and their effects, which led to an overestimation of the measured outcome.

To address these limitations, the present study systematically evaluates the effectiveness of crossword puzzles in a medical terminology course using sound methodology, appropriate outcome measures (test scores), and rigorous statistical analyses. Specifically, the study pursues two objectives: first, to examine the overall effectiveness of the crossword puzzle intervention among students; and second, to determine the extent to which this intervention enhances test performance among low-performing students who had previously failed to achieve satisfactory quiz scores. By implementing this evidence-based approach, the research aims to provide a more comprehensive understanding of the impact of crossword puzzles on learning outcomes and to contribute to the growing body of literature on gamified educational tools.

## 2 Methods

### 2.1 Study design

This study employs a non-equivalent control group post-test-only design, which is a type of quasi-experimental design. This method is commonly used to compare outcomes between a treatment group and a control group without the need for random assignment (19). The non-equivalent control group post-test-only design was chosen because a pre-post test approach was not suitable for this study. Administering a pre-test on medical terminology would have exposed both groups to the same test items, potentially triggering a “testing effect” that could artificially enhance students’ familiarity with the content and bias the post-test results. Moreover, because the test items used in this study were drawn from the actual final examination that contributes to students’ cumulative grade point average (CGPA), administering them beforehand could have compromised the integrity of the course assessment. In addition, since the course content was sequentially taught throughout the semester, introducing a pre-test might have prematurely revealed key concepts, thereby influencing students’ learning trajectory and diminishing the authenticity of the intervention’s impact. The post-test-only design avoids these threats by ensuring that any observed differences in test scores can be more confidently attributed to the crossword puzzle intervention rather than to pre-test sensitization, compromised assessment integrity, or unintended instructional cues. In this study, the treatment group consisted of students who utilized paper-based crosswords as a learning aid, while the control group did not receive this intervention. After the intervention, both groups completed the final test, and their scores were compared to assess the impact of the crossword puzzle activity on learning outcomes.

### 2.2 Course information

The Medical Terminology course (HSM544) is a core requirement for the bachelor’s degree in health administration, a three-year undergraduate program. Offered in Year 2 (Semester 3), this course is compulsory for graduation and serves as a prerequisite for advanced courses such as Medical Coding and Epidemiology, which are available in Semesters 4 and 5. The course carries 4 credit hours and consists of 3 contact hours per week over a 14-week semester. It is delivered through face-to-face lectures, allowing direct engagement between students and instructors. Student assessment is based on a combination of written assessments, group projects, and online team-based learning. The written assessments include a quiz (20%) in Week 4 and a final test (30%) in Week 14. Additionally, students participate in a group project (30%) and online team-based learning (20%). Students must obtain at least 50% of the total continuous assessment marks (Grade C or higher) to pass the course. The course content covers essential medical terminology, beginning with an introduction to medical terminology and the body system, followed by system-based topics such as the musculoskeletal, cardiovascular, respiratory, nervous, integumentary, and reproductive systems, as well as the sense organs (eye and ear). For each body system, lectures emphasize terminology related to anatomical structures and functions, pathology, common diagnoses, and treatment approaches. This course provides students with a strong foundation in medical vocabulary, which is essential for their academic progression and future roles in health administration. All course materials, including lectures, discussions, and assessments, were conducted entirely in English.

### 2.3 Study participants and subject selection

**Figure 1:**
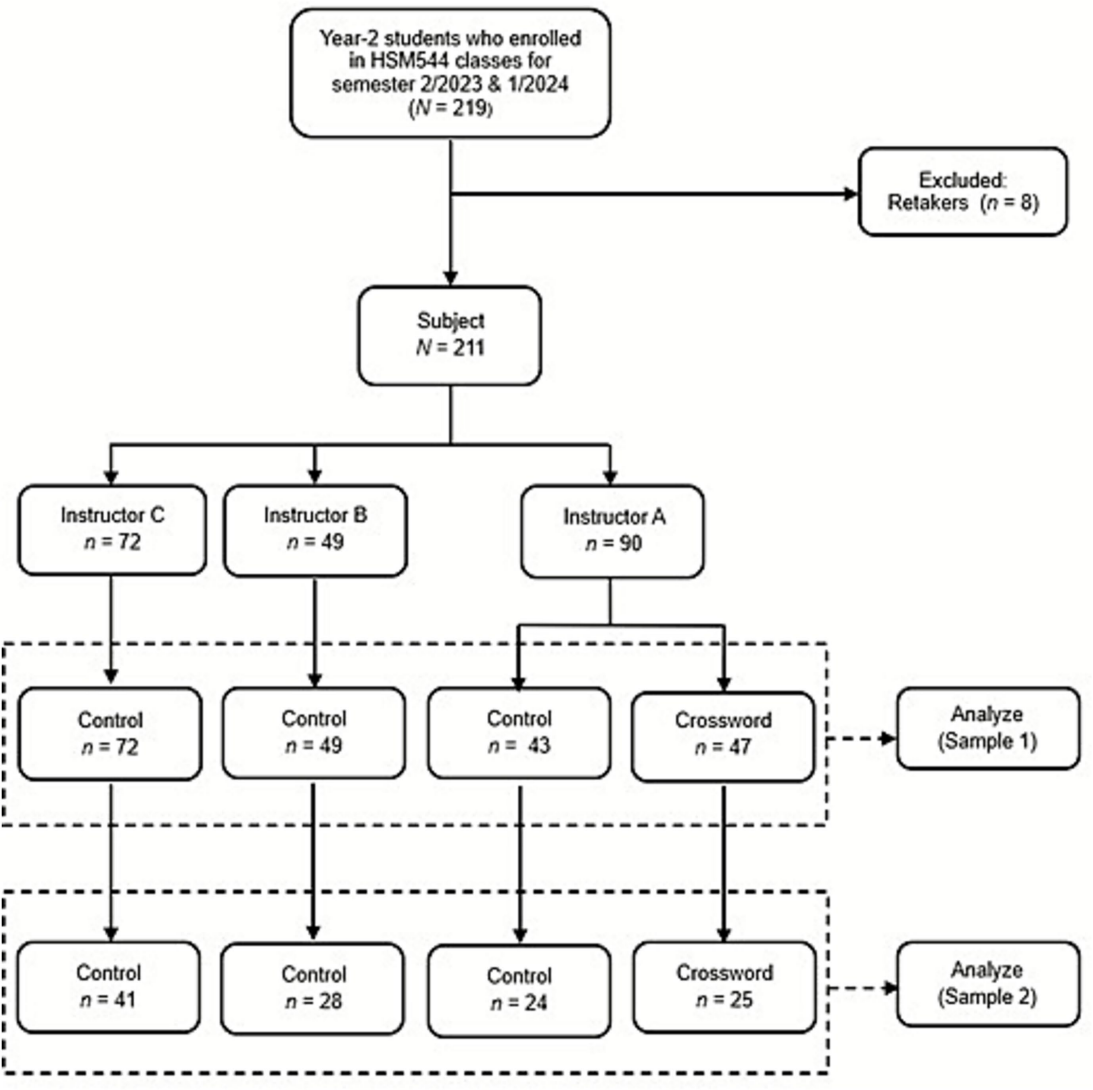
Study participants and subject selection.

A total of 211 Year-2 students enrolled in the Medical Terminology (HSM544) course during Semester 2/2023 and Semester 1/2024 were selected for this study. Students who had previously failed and were retaking the course (*n* = 8) were excluded. These students were distributed across eight class time slots, taught by three instructors: Instructor A (4 classes, *n* = 90), Instructor B (2 classes, *n* = 49), and Instructor C (3 classes, *n* = 72). For the study, two of Instructor A’s classes were assigned as the treatment group (*n* = 47), where students used crosswords as a learning aid, while the remaining students formed the control group (*n* = 164). In Analysis 1, the final test scores between these two groups were compared to evaluate the overall impact of the intervention. Additionally, participants were resampled based on their quiz performance to examine the effect of crosswords on non-performing students. In Analysis 2, only students who scored below 50% on the quiz were selected, leading to a comparison between the control group (*n* = 93) and the intervention group (*n* = 25) based on their final test scores. Figure 1 shows the flowchart of study participants and subject selection.

### 2.4 Crossword as intervention

The intervention outlined in this study involved the use of crossword puzzles as learning aids for students in two selected classes. Crossword puzzles were created using a free online puzzle maker.^1^ In accordance with the platform’s terms of use, instructors were permitted to generate and distribute the printed worksheets for private classroom use.

For each lecture, the instructors selected 20 medical terms aligned with the lecture content to serve as crossword clues. A combination of complete sentence definitions and fill-in-the-blank prompts was used to facilitate learning and ensure consistency in cognitive demand across sessions. All crossword materials were developed by the research team based on the course syllabus and were reviewed prior to implementation to ensure content relevance and comparable difficulty levels throughout the intervention period.

This intervention commenced in teaching week 5 and continuously over a nine-week period. Prior to the start of the intervention, all participating students received a standardized briefing explaining the purpose, procedures, and expectations of the crossword activities. Informed consent was obtained from all participants before implementation. To ensure intervention fidelity, the same procedures, materials, and delivery format were applied consistently in every lecture. The intervention was administered by the course instructor following a predefined protocol, including standardized instructions, fixed timing, and uniform incentives.

On the day of each lecture, printed copies of a crossword puzzle (Set A) were distributed to all students. While listening to the instructor’s lecture or viewing instructor-selected educational videos, students were required to complete the crossword puzzle concurrently. At the end of the session, students submitted their completed or partially completed Set A crosswords, allowing the instructor to verify participation and adherence to the activity. Immediately after submission, a second crossword puzzle (Set B), covering the same content but presented in a different layout, was distributed. Students were instructed to complete this puzzle as quickly as possible, with a timer used to record completion times. To reinforce engagement, a small cash incentive was awarded to the two students who completed the puzzle in the shortest time. Students were then allowed to take Set B home and were required to upload a scanned copy to the course portal for documentation purposes.

Student compliance was monitored through a combination of in-class attendance records, submission of Set A crosswords during each lecture, and online uploads of Set B crosswords. Participation logs were maintained across all sessions to track individual student engagement. For inclusion in the final analysis, students were required to participate in at least 80% of the intervention sessions, defined as attendance and submission of the required crossword activities. This monitoring approach ensured that outcome analyses reflected adequate exposure to the intervention and supported the internal validity of the study.

### 2.5 Study variables

The study variables include sex, current cumulative grade point average (CGPA), entrance qualification, entrance CGPA, academic stream, and Malaysian University English Test (MUET) results. Current CGPA refers to the average grade points achieved by a student across all courses taken in previous semesters. This institution uses a 4.0 grading system, where the maximum possible CGPA is 4.0, representing consistently excellent performance. Current CGPA is categorized into five groups: less than 3.00, 3.00 to 3.32, 3.33 to 3.49, 3.50 to 3.67, and 3.68 or higher. Entrance qualification refers to any formal qualification that students use to gain entry into the current undergraduate program. This three-year undergraduate program accepts high school certificates and their equivalents (e.g., pre-university certificates, foundation program certificates) as part of its admission requirements. Admissions from other pathways, such as three-year undergraduate diplomas, are also common, with credit transfers allowed for first-year courses. This program is open to students from all academic disciplines, which are generally classified into science, technology, engineering, and mathematics (STEM), as well as others (non-STEM), including business, management, arts, and social sciences. Entrance CGPA refers to the average grade points obtained by students in their entrance qualifications. Like the current CGPA, the entrance CGPA is also grouped into five categories. The Malaysian University English Test (MUET) is a standardized test that assesses English language proficiency, and the results are required for admission into this undergraduate program. The results are represented by bands, which indicate proficiency, ranging from Band 1 (limited user) to Band 5 + (highly proficient user). In this study, MUET results are classified into four categories: less than Band 3, Band 3.0 to 3.5, Band 4.0, and Band 4.5 to 5+.

### 2.6 Study outcomes

The primary outcome measured in this study is the final test score, which assesses the effect of using crossword puzzles as learning aids. The test was conducted at the end of Week 14 of the teaching semester and lasted 2 h. The test paper consisted of three sections: Term-Building (T-B), Term-Defining (T-D), and Short Answer Question (SAQ), with a total possible score of 80 marks. In Part A (Term-Building), students were required to construct medical terms based on the descriptions provided. This section contained 20 descriptions, with one mark awarded per correct answer, for a total of 20 marks. Part B (Term-Defining) required students to define specific medical terms, particularly those that were compounded (e.g., myasthenia gravis) or could not be easily defined by word components (e.g., anaemia). This section included 10 terms, with two marks awarded per correct definition, making a total of 20 marks. Part C (Short Answer Questions) consisted of four questions, each with two sub-questions (A and B). Sub-question A required students to answer in a listing format, while sub-question B required a brief explanation. Each question carried 10 marks, contributing to a total of 40 marks. To analyze the impact of crossword usage, we examined the effect on individual test components (T-B, T-D, and SAQ) as well as the Total Score (TS). Students were required to answer all questions in English. After the test concluded, the answer sheets and question papers were collected from the students. To maintain consistency and fairness in the evaluation process, a collaborative marking scheme was implemented, allowing all instructors to work together to assess and grade the papers. To simplify interpretation, the marks obtained for each component and the total score were converted to a scale of 100 points for analysis.

### 2.7 Psychometric properties of the final test question paper

Analyses of the final test question paper indicated overall satisfactory psychometric properties. Both the 2-parameter logistic (2PL) model and the Graded Response Model (GRM) showed that items in Parts A, B, and C demonstrated acceptable levels of discrimination, ranging from moderate to high, and were generally of moderate difficulty, with no evidence of items being excessively easy or difficult. The test characteristic curves (TCCs) further suggested that students performing above average were likely to attain reasonable scores across different tasks, such as constructing, defining, and explaining medical terms. Reliability analyses using Kuder–Richardson’s test (KR-20), Cronbach’s alpha, and the Person Separation Index (PSI) consistently confirmed good internal consistency of the items, and interrater reliability measures, including percent agreement, Krippendorff’s alpha, and intraclass correlations, indicated strong agreement among raters, supporting the robustness of the scoring process. The details of these analyses and their results are provided in the Supplementary File.

### 2.8 Statistical analysis

In this study, categorical variables were analyzed using frequency and percentage, while continuous variables were analyzed using mean, standard deviation, and standard errors. The statistical analysis began with descriptive analyses to compare the profiles of students in the control and intervention groups. The examined profiles included sex, current cumulative grade point average (CGPA), entrance qualification, academic stream before enrollment, entrance CGPA, and the Malaysian University English Test (MUET) results. To determine significant differences in categorical characteristics between groups, likelihood-ratio chi-squared tests were conducted.

For the primary analysis, the marks for each final test component and total score were compared between the control and intervention groups using an independent *t*-test. Welch’s approximation was applied if the assumption of equal variance was violated. To account for potential confounders, marginal mean scores, adjusted for subject covariates, were computed using multiple linear regression with robust variance estimates.

To examine the causal effect of the intervention on the measured outcomes, the researcher conducted treatment-effect analyses. This process began by matching each student in the intervention group with two students in the control group (1:2 ratio) based on the propensity score of matching covariates. A greedy matching algorithm with a caliper set at 0.20 was used to compute the propensity scores and identify matched subjects (20, 21). Following the matching process, treatment effect analysis was conducted using the user-written module “treatrew,” which estimates the average treatment effect (ATE), average treatment effect for the treated (ATET), and average treatment effect for the non-treated (ATENT) by re-weighting the propensity score estimator (22). Additionally, a sensitivity analysis was performed to assess the presence of unobserved bias in ATET estimation, following the approach recommended by Rosenbaum (23, 24). All reported *p*-values were two-tailed, and the significance level was set at *p* < 0.05. Stata Statistical Software: Release 18 (StataCorp LP, College Station, TX, USA) was used to analyze the data.

## 3 Results

### 3.1 Baseline characteristics

The demographic and academic profiles of the study participants were analyzed and compared between two groups: the intervention group, consisting of students who used crosswords as learning aids, and the control group, which did not receive this intervention. This comparison aimed to determine whether any significant differences existed between the groups in terms of demographic characteristics and academic performance indicators. In the first sample (Sample 1), which included 211 students, statistically significant differences were observed in the distribution of students across different current CGPA categories (LR *χ*^2^ (4) = 17.89, *p* = 0.001) and entrance CGPA categories (LR *χ*^2^ (4) = 10.35, *p* = 0.035). Specifically, a higher proportion of students with high current CGPAs and high entrance CGPAs was found in the intervention group compared to the control group. However, no statistically significant differences were observed between the intervention and control groups when examining other demographic and academic variables. These variables included sex, entrance qualification, academic stream, and the university’s English test scores, indicating that these factors were evenly distributed between the two groups.

A separate analysis was conducted on Sample 2, which comprised 118 students who had previously scored less than 50% on quizzes. In this subset, no significant differences were found between the intervention and control groups across all examined variables, including sex, current CGPA, entrance qualification, academic stream, entrance CGPA, and university English test scores. This suggests that student characteristics within this lower-performing subgroup were evenly distributed between those who participated in the crossword-based intervention and those who did not. Table 1 presents a comprehensive summary of the demographic and academic profiles of the students included in this study.

**Table 1:** Demographic and academic profiles of students enrolled in control and intervention (crossword) groups.

Variable	ALL		Sample 1				*p*	Sample 2				*p*
	(*N* = 211)		Control (*n* = 164)		Crossword (*n* = 47)			Control (*n* = 93)		Crossword (*n* = 25)		
	*F*	%	*F*	%	*F*	%		*F*	%	*F*	%	
Sex												
Male	25	11.9	21	12.8	4	8.5	0.42	12	12.9	3	12.0	0.90
Female	186	88.2	143	97.2	43	91.5		81	87.1	22	88.0	
Current CGPA												
< 3.00	14	6.6	12	7.3	2	4.3	0.00	9	9.7	1	4.1	0.05
3.00–3.32	58	27.5	51	31.1	7	14.9		32	34.4	6	24.0	
3.33–3.49	66	31.3	55	33.5	11	23.4		35	37.6	7	28.0	
3.50–3.67	60	28.4	41	25.0	19	40.4		16	17.2	8	32.0	
≥ 3.68	13	6.2	5	3.1	8	17.0		1	1.1	3	12.0	
Entrance Qual.												
HS certificate	21	10.0	19	11.6	2	4.3	0.19	12	12.9	2	8.0	0.65
Pre-u certificate	52	24.6	37	22.6	15	32.0		10	10.8	4	16.0	
Diploma	138	65.4	108	65.9	30	63.8		71	76.3	19	76.0	
Academic stream												
Non-STEM	117	55.5	95	57.9	22	46.8	0.17	65	68.9	15	60.0	0.35
STEM	94	44.6	69	42.1	25	53.2		28	30.1	10	40.0	
Entrance CGPA												
< 3.00	20	9.5	19	11.6	1	2.1	0.04	15	16.1	1	4.0	0.08
3.00–3.32	49	23.2	41	25.0	8	17.0		29	31.2	4	16.0	
3.33–3.49	26	12.3	21	12.8	5	10.6		16	17.2	4	16.0	
3.50–3.67	65	30.8	50	30.5	15	31.9		20	21.5	10	40.0	
≥ 3.68	51	24.2	33	20.1	18	38.3		13	14.0	6	24.0	
MUET												
< 3.0	10	4.7	10	6.1	0	0	0.12	9	9.7	0	0	0.06
3.0–3.5	87	41.2	71	43.3	16	34.0		45	48.4	10	40.0	
4.0	93	44.1	69	42.1	24	51.1		36	38.7	12	48.0	
≥ 4.5	21	10.0	14	8.5	7	14.9		3	3.2	3	12.0	

CGPA, Cumulative Grade Point Average; STEM, Science, Technology, Engineering, and Mathematics.

*p*-values were based on Likelihood-ratio chi-squared test.

### 3.2 Mean comparison

Independent *t*-tests were conducted to compare the scores obtained on the final test. The analysis focused on examining the mean scores for each test component: term-building (T-B), term-defining (T-D), short answer questions (SAQs), and the total score (TS) for the test.

In Sample 1, the results indicated that, on average, students who used crosswords earned 58 points for T-B, 66 points for T-D, and 73 points for SAQs, contributing a total score of 67 points for the final test. These scores translate to an average of 11 correctly built medical terms, 6 correctly defined medical terms, and more than two SAQs answered accurately. In contrast, students who did not use crosswords earned significantly fewer points on average: 24 points for T-B, 23 points for T-D, 48 points for SAQs, and a total score of 37 points. This translates to about 5 correctly built medical terms, 2 correctly defined medical terms, and fewer than two SAQs answered correctly. The largest mean score difference was observed in the term-defining component, with an estimate of −43.27 (*SE* 4.41, 95% CI: −51.97, −34.57).

Subsequent analyses of Sample 2 also revealed a significant improvement in student’s performance on the final test. On average, students who received the intervention scored 41 points for T-B, 54 points for T-D, and 73 points for SAQ. These scores correspond to an average of four correctly constructed medical terms, five accurately defined medical terms, and more than two completely answered short-answer questions. In contrast, students in the control group managed to construct and define fewer than three medical terms and correctly answered fewer than two short answer questions. Similar to Sample 1, the largest mean difference was observed in the term-defining component, with an estimate of −42.14 (*SE* 6.31, 95% CI: −55.07 to −29.23).

Improvements in total scores were observed among students who used crosswords as learning aids in both samples, and these findings were statistically significant. In Sample 1, the estimated mean difference was −32.36 (*SE* 2.06, 95% CI: −36.46 to −28.27), while in Sample 2, it was −33.06 (SE 1.84, 95% CI: −36.72 to −29.39). These substantial differences indicate that the intervention significantly enhances overall performance on the final test. The calculated Hedges’s *g* indicated that the effect sizes of these statistically significant findings are greater than 1, suggesting that the differences exceed one standard deviation. Such large effect sizes signal a substantial impact, carrying strong practical implications. Table 2 summarizes the results of the independent t-tests conducted on both samples.

**Table 2:** Result of mean comparison analysis according to final test components.

Variable	Sample 1						Sample 2					
	*M*	*SE*	*t*	*df*	*p*	*g*	*M*	*SE*	*t*	*df*	*p*	*g*
T-B												
Control	24.25	1.99	-6.48^a^	63.50^a^	<0.001	−1.22	13.23	1.75	−3.90^a^	27.49^a^	<0.001	−1.26
Cross	57.92	4.79					40.80	6.85				
$\bar{D}$	−33.68	5.19					−27.57	7.07				
T-D												
Control	22.68	2.08	−9.81	209	<0.001	−1.62	12.15	1.74	−6.68^a^	28.38^a^	<0.001	−2.06
Cross	65.96	3.90					54.30	6.06				
$\bar{D}$	−43.27	4.41					−42.15	6.31				
SAQ												
Control	47.89	1.42	−8.73	209	<0.001	−1.44	43.30	1.90	−7.45	116	<0.001	−1.66
Cross	73.34	2.18					72.76	2.85				
$\bar{D}$	−25.44	2.91					−29.46	3.95				
TS												
Control	37.20	1.28	−15.69^a^	112.90^a^	<0.001	−2.08	30.26	1.34	−17.93^a^	85.59^a^	<0.001	−2.76
Cross	69.57	1.61					63.32	1.25				
$\bar{D}$	−32.36	2.06					−33.06	1.84				

T-B, Term-building; T-D, Term-defining; SAQ, Short answer question; TS, Total score.

Score for each component was standardized to 100. $\bar{D}\;$ represents mean difference.

a The t and df were adjusted using Welch’s approximation.

Unmeasured covariates may confound the results of an independent *t*-test. To address this concern, subsequent analyses employed multiple regression models to statistically adjust for several covariates, including sex, current CGPA, admission qualifications, academic stream, entrance CGPA, university’s English test results, and course instructor. Our data violate the assumption of homoscedasticity; thus, robust variance estimates were used. We calculated the marginal (adjusted) means and linear contrasts for each test component, as well as the total score. Table 3 provides a summary of the adjusted means based on the multiple regression analyses, while the full results can be found in Table 4.

**Table 3:** Marginal mean and contrast coefficient based on the results of regression analyses.

Variable	Sample 1					Sample 2				
	*M*	*SE*	*t*	*95% CI*	*p*	*M*	*SE*	*t*	*95% CI*	*p*
T-B										
Control	25.33	2.00	12.64	21.38–29.28	<0.001	12.44	1.90	6.56	8.68–16.19	<0.001
Crossword	54.15	5.29	10.24	43.72–64.59	<0.001	43.74	8.04	5.44	27.80–59.8	<0.001
Contrast (ψ)	28.82	6.11		16.76–40.88	<0.001	31.30	8.85		13.76–48.85	<0.001
T-D										
Control	24.27	2.11	12.48	20.11–28.44	<0.001	12.09	1.94	6.24	8.25–15.93	<0.001
Crossword	60.40	4.49	13.45	51.55–69.25	<0.001	54.54	6.57	8.30	41.51–67.56	<0.001
Contrast (ψ)	36.12	5.46		25.35–46.90	<0.001	42.45	7.19		28.19–56.71	<0.001
SAQ										
Control	49.37	1.37	36.09	46.67–52.07	<0.001	44.52	2.02	22.05	40.52–48.52	<0.001
Crossword	68.22	2.47	27.67	63.36–73.08	<0.001	68.23	3.36	20.29	61.57–74.90	<0.001
Contrast (ψ)	18.85	3.09		12.75–24.94	<0.001	23.71	4.48		14.84–32.58	<0.001
TS										
Control	38.67	1.19	32.47	36.32–41.02	<0.001	30.79	1.46	21.12	27.90–33.68	<0.001
Crossword	64.46	1.97	32.79	60.58–68.33	<0.001	61.36	2.53	24.25	56.35–66.38	<0.001
Contrast (ψ)	25.78	2.61		20.64–30.93	<0.001	30.57	3.37		23.89–37.25	<0.001

T-B, Term-building; T-D, Term-defining; SAQ, Short answer question; TS, Total score.

The score for each component was standardized to 100.

The regression models were adjusted for gender, current CGPA, admission route, academic stream, entrance CGPA, English proficiency test result (MUET – Malaysian University’s English Test), and instructor.

**Table 4:** Summary of the results of regression analysis.

Variable	Term-building					Term-defining					Short answer question					Total score				
	Coef.	*SE*	*t*	*p*	η^2^	Coef.	*SE*	*t*	*p*	η^2^	Coef.	*SE*	*t*	*p*	η^2^	Coef.	*SE*	*t*	*p*	η^2^
Sample 1 (*n* = 211)																				
Model					0.38					0.45					0.44					0.62
CROSS	28.82	6.11	4.71	<0.001	0.14	36.12	5.46	6.61	<0.001	0.20	18.85	3.09	6.10	<0.001	0.14	25.78	2.61	9.88	<0.001	0.32
Sex	15.05	4.28	3.51	0.001	0.04	5.98	5.36	1.12	0.266	0.01	5.11	3.57	1.43	0.154	0.01	7.56	2.35	3.22	0.002	0.04
cCGPA	−1.84	2.24	−0.82	0.413	0.00	3.08	1.86	1.65	0.100	0.01	5.75	1.09	5.29	<0.001	0.10	3.88	1.00	3.89	<0.001	0.07
eQual	−1.19	2.15	−0.55	0.580	0.00	−2.46	2.46	−1.00	0.319	0.00	−1.32	1.74	−0.76	0.448	0.00	−1.56	1.32	−1.18	0.238	0.01
STEM	15.62	3.66	4.27	<0.001	0.08	12.10	3.61	3.35	0.001	0.06	−2.90	2.12	−1.34	0.172	0.01	3.48	1.83	1.90	0.059	0.02
eCGPA	3.71	1.30	2.85	0.005	0.03	2.97	1.30	2.28	0.024	0.02	1.04	1.04	1.00	0.317	0.01	1.69	0.77	2.20	0.029	0.02
MUET	7.64	2.61	2.92	0.004	0.04	7.37	2.64	2.79	0.006	0.04	5.42	1.61	3.36	0.001	0.05	6.46	1.33	4.86	<0.001	0.10
INST	0.67	2.21	0.30	0.763	0.00	0.26	2.42	0.11	913	0.00	−0.70	1.52	−0.46	0.647	0.00	−0.43	1.27	−0.34	0.737	0.00
Cons.	−19.93	10.82	−1.84	0.067		−18.95	10.88	−1.74	0.083		16.07	7.94	2.02	0.044		1.17	5.78	0.20	0.840	
*F*	(8, 202) 19.13			<0.001		(8, 202) 27.93			<0.001		(8, 202) 26.38			<0.001		(8, 202) 57.21			<0.001	
*R* ^2^	0.38					0.45					0.44					0.62				
RMSE	24.66					24.28					15.605					12.87				
Sample 2 (*n* = 118)																				
Model					0.36					0.46					0.45					0.62
CROSS	31.30	8.85	3.54	0.001	0.21	42.45	7.19	5.90	<0.001	0.33	23.71	4.48	5.30	<0.001	0.19	30.57	3.37	9.06	<0.001	0.43
Sex	11.66	5.39	2.16	0.033	0.04	0.34	6.56	0.05	0.959	0.00	−3.21	4.71	−0.68	0.497	0.00	1.48	2.99	0.49	0.622	0.00
cCGPA	−7.14	2.97	−2.40	0.018	0.10	−2.37	2.08	−1.14	0.255	0.01	5.54	1.59	3.50	0.001	0.09	1.40	1.15	1.21	0.229	0.01
eQUAL	1.65	2.42	0.69	0.495	0.00	0.81	2.23	0.37	0.716	0.00	−4.61	2.56	−1.80	0.075	0.03	−2.16	1.61	−1.34	0.183	0.02
STEM	6.97	4.56	1.53	0.129	0.02	6.87	4.79	1.43	0.155	0.02	−3.08	3.26	−0.94	0.348	0.01	0.09	2.43	−0.04	0.969	0.00
eCGPA	2.96	1.46	2.02	0.045	0.03	2.63	1.44	1.83	0.070	0.02	1.64	1.43	1.15	0.253	0.01	1.65	0.98	1.69	0.094	0.03
MUET	0.22	3.18	0.07	0.945	0.00	0.16	3.06	0.05	0.960	0.00	4.57	2.56	1.78	0.077	0.03	3.21	1.73	1.85	0.067	0.03
INST	2.38	2.30	1.03	0.304	0.01	1.47	2.76	0.54	0.594	0.00	−0.09	2.35	−0.04	0.971	0.00	0.60	1.76	0.34	0.734	00
Cons.	1.43	9.73	0.15	0.884		2.84	11.51	0.25	0.806		29.16	9.43	3.23	0.003		17.34	5.61	3.09	0.003	
*F*	(8, 109) 8.06			<0.001		(8, 109) 9.67			<0.001		(8, 109) 16.79			<0.001		(8, 109) 38.55			<0.001	
*R* ^2^	0.36					0.46					0.45					0.62				
RMSE	20.19					20.22					16.34					11.56				

CROSS, Crossword (group); cCGPA, Current Cumulative Grade Point Average; eQUAL, Entrance Qualification; STEM, Science, Technology, Engineering and Mathematics (yes, no); eCGPA, Entrance Cumulative Grade Point Average; MUET, Malaysian University English Test; INST, Instructor.

Based on the results presented in Table 3, students in the intervention group demonstrated higher average scores than those in the control group. Analysis of Sample 1 indicates that students who did not use crosswords as learning aids, on average, scored 25 points for T-B, 24 points for T-D, and 49 points for SAQ. These scores correspond to fewer than six correctly constructed medical terms, fewer than three accurately defined complex medical terms, and fewer than two correctly answered short-answer questions. In comparison, students who utilized crosswords as learning aids had higher adjusted mean scores of 54 points for T-B, 60 points for T-D, and 68 points for SAQ. These differences were associated with a greater number of correctly constructed terms, accurately defined terms, and correct short-answer responses. Specifically, students constructed an additional five correct terms, accurately defined four additional medical terms, and provided one or more correct responses to the short-answer questions.

A similar pattern was observed in Sample 2. Students who utilized crosswords exhibited higher adjusted mean scores across all test components relative to those in the control group. On average, students in the intervention group correctly constructed approximately eight medical terms, accurately defined more than five medical terms, and answered more than two short-answer questions correctly. In contrast, students in the control group constructed fewer than three medical terms, defined fewer than two medical terms accurately, and partially answered approximately one short-answer question correctly.

**Figure 2:**
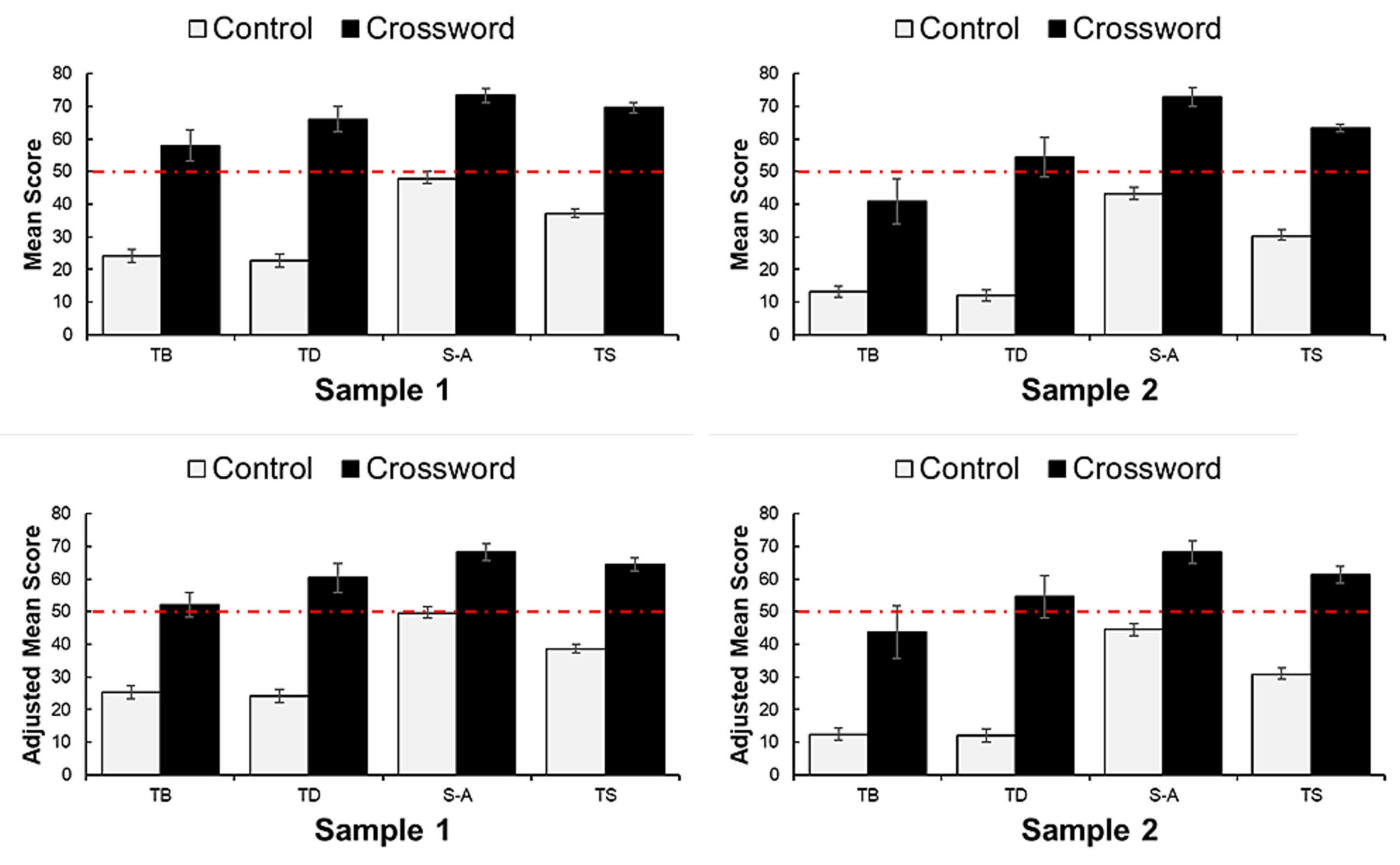
Bar graphs with error bars (standard errors) showing the mean and adjusted mean scores for each assessed component for sample 1 and sample 2.

Across both samples, higher adjusted mean total scores were observed among students who used crossword puzzles. In Sample 1, the adjusted mean total score differed by 26 points between the intervention and control groups, while in Sample 2, the corresponding difference was 31 points. These findings indicate a consistent association between the use of crossword puzzles as learning aids and higher test performance. Figure 2 illustrates performance across individual test components and total scores for both groups, based on independent *t*-tests and multiple regression analyses.

### 3.3 Treatment effect analysis

Treatment effect analysis is commonly used in medical research to estimate differences in outcomes associated with exposure to a specific treatment. While *t*-tests focus on mean differences, treatment effect models allow for a more nuanced examination of outcome differences after accounting for observed confounding variables. Given the non-randomized and observational design of the study, propensity score matching was applied to balance observed covariates between intervention and control groups using a 1:2 matching ratio. In Sample 1, 2 matched controls were identified for 38 intervention participants, and one matched control for six participants, yielding a match sample of 127 students (44 intervention, 83 control). In Sample 2, two matches were identified for 23 intervention participants and one match for five participants, resulting in a total matched sample of 64 students (23 intervention, 41 control). Hosmer-Lemeshow tests indicated adequate model fit for both Sample 1 [*χ*^2^ (8) = 5.86, *p* = 0.663] and Sample 2 [*χ*^2^ (8) = 2.20, *p* = 0.974], suggesting no evidence of non-linearity or interaction effects between confounders and treatment assignment. Additionally, negligible standardized differences across covariates indicated satisfactory balance between matched groups.

Table 5 summarizes the estimates of treatment effects for both samples. The average treatment effect (ATE) represents the average difference in outcomes associated with crossword use across the matched population. In Sample 1, positive ATE estimates were observed across all test components: Term-building (ATE 27.50, 95%CI 16.88–38.11, *p* < 0.001), term-defining (ATE 35.69, 95%CI 25.77–45.61, *p* < 0.001), and short answer questions (ATE 18.88, 95%CI 14.53–25.23, *p* < 0.001). The estimated difference in total test scores associated with crossword use was 26.30 points (95% CI 21.75–30.84, *p* < 0.001). Comparable patterns were observed in Sample 2, where students who used crosswords exhibited higher scores across all assessed components, with an estimated total score difference of 32.46 (95% CI 25.23–39.70, *p* < 0.001).

**Table 5:** The result of treatment effect analyses based on propensity score-matched samples (propensity score reweighted).

Variable	Sample 1 (*n* = 127)					Sample 2 (*n* = 64)				
	Coef.	*SE*	*z*	*95% CI*	*p*	Coef.	*SE*	*z*	*95% CI*	*p*
Term-building										
ATE	27.50	5.42	5.08	16.88–38.11	<0.001	32.45	7.66	4.23	17.42–47.49	<0.001
ATET	27.81	15.79	1.76	−3.14–58.78	0.078	30.56	18.24	1.68	−5.20–66.32	0.094
ATENT	27.32	9.02	3.03	9.63–45.02	0.002	33.52	14.50	2.31	5.09–61.94	0.021
Term-defining										
ATE	35.69	5.06	7.05	25.77–45.61	<0.001	42.40	6.46	6.56	29.73–55.06	<0.001
ATET	34.85	14.63	2.38	6.17–63.52	0.017	41.09	18.04	2.28	5.73–76.44	0.023
ATENT	36.13	9.14	3.95	18.20–54.06	<0.001	43.13	12.92	3.34	17.80–68.46	0.001
Short answer										
ATE	18.88	2.73	7.28	14.53–25.23	<0.001	26.38	3.93	6.72	18.68–34.08	<0.001
ATET	19.52	7.36	2.65	5.09–33.95	0.008	27.02	10.07	2.68	7.28–46.77	0.007
ATENT	20.07	5.18	3.87	9.91–30.22	<0.001	26.02	8.64	3.01	9.08–42.95	0.003
Total score										
ATE	26.30	2.32	11.33	21.75–30.84	<0.001	32.46	3.69	8.80	25.23–39.70	<0.001
ATET	25.94	7.05	3.68	12.13–39.75	<0.001	32.21	9.48	3.40	13.63–50.79	0.001
ATENT	26.48	4.96	5.34	16.78–36.20	<0.001	32.61	8.96	3.64	15.05–50.16	<0.001

ATE, Average Treatment Effect; ATET, Average Treatment Effect on the Treated; ATENT, Average Treatment Effect on the Non-treated.

The score for each component was standardized to 100.

Sample 1 consisted of 44 students in the crossword group matched with 83 students in the control group.

Sample 2 consisted of 23 students in the crossword group matched with 41 students in the control group.

Matching uses a caliper of 0.20. Covariates matched include sex, current CGPA, entrance qualification, academic stream, entrance CGPA, and English proficiency test result (MUET – Malaysian University English Test).

The average treatment effect on the treated (ATET) represents outcome differences associated with crossword use among students who actually used the learning aid. In Sample 1, statistically significant ATET estimates were observed for Term-Building (ATET 34.85, 95% CI 6.17–63.75, *p* = 0.017) and Short Answer Questions (ATET 19.52, 95% CI 5.09–33.95, *p* = 0.008), with a total score difference of 25.94 points (95% CI 12.13–39.75, *p* < 0.001). In Sample 2, significant ATET estimates were observed for Term-Defining (ATET 41.09, 95% CI 5.73–76.44, *p* = 0.023) and short answer questions (ATET 27.02, 95% CI 7.28–46.77, *p* = 0.007), with an estimated difference of 32.21 points in total scores (95% CI 13.63–50.79, *p* = 0.001). No statistically significant ATET estimates were observed for Term-Building in Sample 2.

The Average Treatment Effect on the Non-Treated (ATENT) reflects estimated outcome differences for control-group students under a hypothetical scenario in which they had used crosswords. In Sample 1, positive ATENT estimates were observed across all test components: Term-building (ATENT 27.32, 95%CI 9.63–45.02, *p* = 0.002), term-defining (ATENT 36.13, 95%CI 18.20–54.06, *p* < 0.001), and short answer questions (ATENT 20.07, 95%CI 9.91–30.22, *p* < 0.001), with an estimated total score difference of 26.48 points (95% CI 16.78–3,620, *p* < 0.001). Similar patterns were observed in Sample 2, where ATENT estimates indicated higher scores across all components, with a total score difference of 23.61 (CI 15.05–50.16, *p* < 0.001).

**Figure 3:**
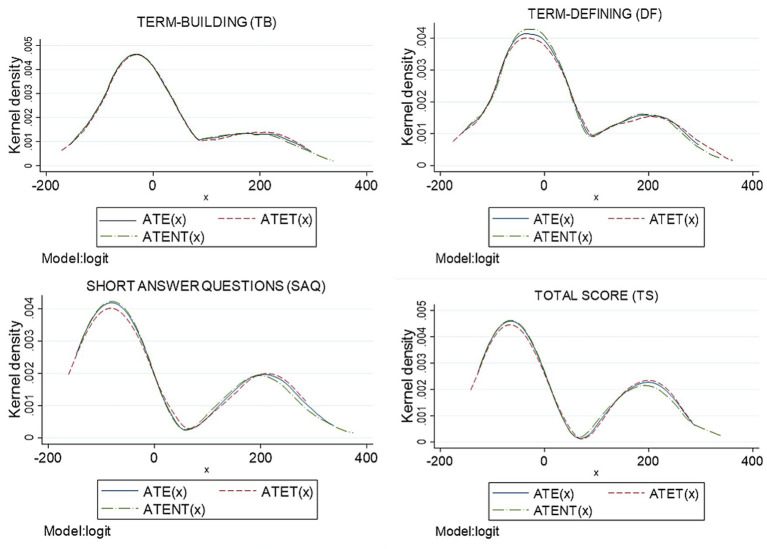
Estimation of the distribution of ATE(*x*), ATET(*x*), and ATENT(*x*) for sample 1 (*n* = 127) by reweighting on the propensity score estimator with a range equal to (−200; 400).

**Figure 4:**
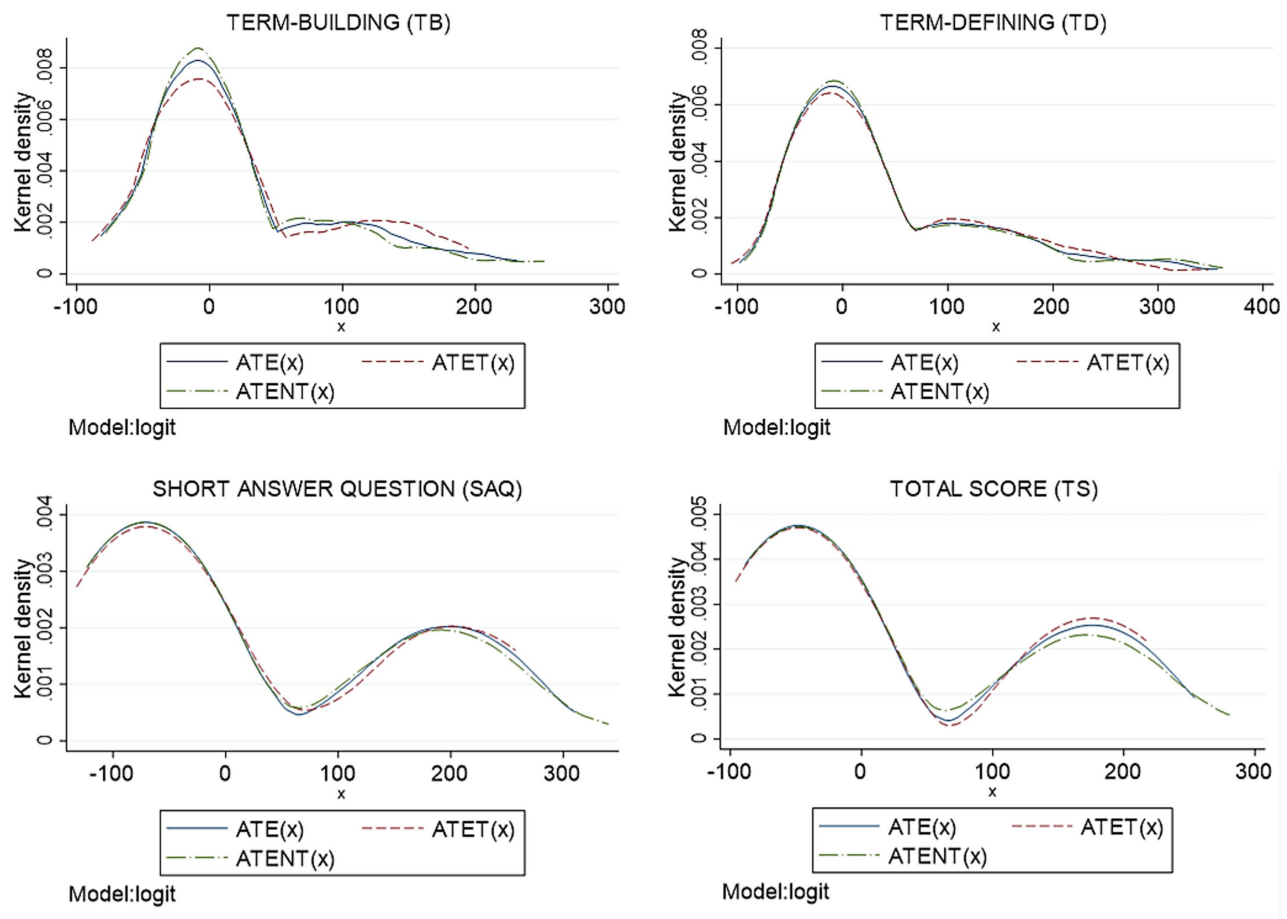
Estimation of the distribution of ATE(*x*), ATET(*x*), and ATENT(*x*) for sample 2 (*n* = 64) by reweighting on the propensity score estimator with a range equal to (−100, 400).

The distributions of individual ATE(x), ATET(x), and ATENT(x) estimated were examined across observed covariates, including sex, current CGPA, entrance qualification, academic stream, entrance CGPA, and MUET results. Figure 3 presents the density distributions for Sample 1, while Figure 4 displays the corresponding distributions for Sample 2. In both samples, the distributions of ATE(x), ATET(x), and ATENT(x) were closely aligned, with ATE(x) and ATENT(x) exhibiting greater concentration at higher values. These patterns suggest that higher test scores were consistently associated with crossword use across observed subgroups.

### 3.4 Sensitivity analysis

Sensitivity analyses were carried out to detect hidden biases that might alter the qualitative conclusions of our findings. Using Rosenbaum’s procedure for bounding the treatment effect estimates, we calculated the Wilcoxon sign-rank test *p*-value for the average treatment effect on the treated while setting the level of hidden bias to a certain value of *Γ*, which reflects the assumption about unmeasured heterogeneity or endogeneity in treatment assignment expressed in terms of the odds ratio of differential treatment assignment due to an unobserved covariate (24). At each *Γ*, we calculated a hypothetical significance level *p*-critical, which presents the bound on the significance level of the treatment effect in the case of endogenous self-selection into treatment status. Table 6 summarizes the results of the sensitivity analysis performed on each test component and the total score for both Sample 1 and Sample 2. The sensitivity analysis results suggest that the study findings are highly robust to hidden bias. According to Rosenbaum (23, 25), a Γ value greater than 2 generally indicates strong resistance to unmeasured confounding, meaning that an unobserved factor would need to significantly influence treatment assignment to alter the conclusions. With values extending beyond 3.0, and in some cases exceeding 10.0, the likelihood of a hidden confounder nullifying the treatment effect is minimal. These results provide strong support for the validity of the observed effect, reinforcing confidence in the study’s conclusions despite the non-randomized design.

**Table 6:** Result of sensitivity analyses detecting hidden (unobserved) bias.

Variable	Sample 1		Sample 2	
	Γ = 1	Γ, max *p*-value	Γ = 1	Γ, max *p*-value
Term-building	<0.001	3.2, 0.047	<0.001	5.0, 0.044
Term-defining	<0.001	4.0, 0.048	<0.001	6.0, 0.043
Short answer question	<0.001	5.5, 0.049	<0.001	5.5, 0.048
Total score	<0.001	12.1, 0.048	<0.001	6.4, 0.047

## 4 Discussion

The present study suggests an association between the use of crossword puzzles as learning aids and improved test performance. Students who utilized crosswords showed an average increase of 28 points in the term-building section (Part A), 35 points in the term-defining section (Part B), and 19 points in the short-answer section (Part C). This positive association was particularly pronounced among students who had previously underperformed on quizzes. Compared to their counterparts who did not use crosswords, these students exhibited an additional increase of 28 points in the term-building section, 40 points in the term-defining section, and 23 points in the short-answer section. These findings are consistent with the interpretation that crosswords may enhance students’ abilities to construct and define terms, as well as to respond to short-answer questions. This aligns with educational research demonstrating that active, retrieval-based learning tools promote deeper encoding and long-term retention of foundational knowledge (26, 27). Moreover, the observed association was further evidenced by an average increase of 26 points in total scores for students in Sample 1 and 29 points for those in Sample 2.

Several studies have corroborated the positive associations between crossword-based learning and academic performance, particularly in test scores. For instance, Patel and Dave (28) examined the influence of crosswords on exercise physiology scores among undergraduate medical students and reported an overall mark increase of 120%. Similar gains have been documented in pharmacology (29), clinical biochemistry (30), and nursing education (31), where crossword integration correlated with significant improvements in scores on both formative and summative assessments. Additionally, research aimed at enhancing the midwife emergency curriculum revealed substantial gains in both theoretical and practical test scores, with relative learning improvements of 68 and 35%, respectively (32). Other studies employing randomized designs have reported similar findings (1, 10, 33). Notably, Gaikwad and Tankhiwale (33) found an impressive 111.33% relative learning gain in pharmacology test scores among students who incorporated crosswords as a learning tool.

Despite the significant improvements observed across various test components, the increase remains suboptimal for low-performing students (Sample 2) in their ability to accurately construct medical terms based on provided descriptions. This pattern of differential effectiveness mirrors findings from other educational interventions, where supplemental tools often yield more modest gains among students with significant knowledge gaps compared to their higher-performing peers (34, 35). Following the intervention, the average score in this category was only 41 points, equating to correctly constructing 8 out of 20 terms. This outcome falls short of the expected threshold of 50 points or more (at least 10 correctly constructed terms). Therefore, an alternative or supplementary intervention, such as a student-led objective tutorial (SLOT), may be necessary to further enhance learning outcomes. A study comparing the efficacy of crossword puzzles and SLOT as innovative teaching strategies found that students in the SLOT group achieved superior test scores (36). Participants reported that SLOT sessions significantly enhanced their understanding of pharmacological concepts, whereas crossword puzzles were primarily beneficial for memorizing drug names. Given that the test items in our study’s term-building component (Part A) require a high level of comprehension regarding the structural composition of medical terminology, exploring the SLOT intervention as a complementary approach warrants further investigation.

The potential motivational role of incentives should be considered when interpreting these results. While the crossword puzzles were presented as a voluntary learning aid, the context of the course and the potential for improved grades may have served as an extrinsic motivator influencing student engagement with the intervention. As reported in a meta-analysis and systematic review, this motivational component may have contributed to the observed performance gains (37, 38), independent of the pedagogical efficacy of puzzles themselves. The intersection of gamification, motivation, and learning outcomes is well-documented in educational literature (39, 40). Future research would benefit from designs that can disentangle the effects of the learning tool from the motivational influence of associated incentives or perceived academic advantage.

In response to methodological considerations, our presented analyses also evaluated potential instructor or class-level confounding. The variable “instructor” was incorporated into our regression models to statistically adjust for any systematic effects attributable to different instructors or classroom environments. The inclusion of this variable was not statistically significant (*p* > 0.05), and the change in the exposure coefficient between the crude and adjusted models was negligible. Furthermore, its inclusion did not materially alter the prior effect estimates for the crossword puzzle intervention. Subsequent analyses employed propensity score matching (PSM), in which each student who received the intervention was matched to two students from the control group based on specified covariates. However, due to limited sample size and the inability to find sufficient matched subjects within the desired caliper, the “instructor” variable was not included as a matching criterion. When we attempted to re-weight the propensity scores based on the ‘instructor’ variable, this resulted in a substantially reduced sample size (from 127 to 68 for Sample 1, and from 64 to 30 for Sample 2) without producing significant changes in the Average Treatment Effect (ATE), Average Treatment Effect on the Treated (ATET), or Average Treatment Effect on the Non-Treated (ATENT) estimates. Consequently, we elected not to re-weight based on the instructor variable to control for selection and confounding bias, as it introduced substantial inefficiency with little impact on the treatment effect estimates.

While our regression and PSM analyses suggest that instruction-specific factors did not act as major confounders in the observed association between puzzle use and test performance in this study, we acknowledge that our ability to fully model clustered data (students nested within classes) was limited by sample size and design constraints. A more robust multilevel linear model approach, while methodologically preferable for such nested data, was not feasible. Consequently, the potential for unmeasured class-level effects (e.g., differences in teaching style, classroom dynamics, or minor variations in content delivery not capture by the instructor variable) cannot be entirely ruled out as a source of residual confounding.

This study is subject to several limitations that affect the interpretation of its findings. First, the absence of randomization and the use of a post-only intervention design preclude definitive causal conclusions and instead indicate an observed association between the intervention and outcomes, due to the lack of baseline measurements. The non-randomized design is susceptible to selection bias and confounding, which can lead to an overestimation of the treatment effect. To address these concerns, we incorporated students’ covariates as potential confounders and controlled for them in our statistical models. While many studies utilize ANCOVA to mitigate the influence of confounders, violations of statistical assumptions—particularly the homogeneity of regression slopes—rendered this method unsuitable. Instead, we employed linear regression models, which offer greater flexibility and are preferred for analyzing treatment effects in observational, non-randomized data. Furthermore, we applied propensity score matching to mitigate selection bias and confounding effects. This technique approximates the conditions of randomized controlled trials, enabling the estimation of causal treatment effects despite the absence of random assignment. Our treatment effect analyses (Average Treatment Effect, Average Treatment Effect on the Treated, and Average Treatment Effect of the Non-Treated) consistently indicated a significant positive association, except for the effect among treated students in the term-building test component (Part A). It is critical to reiterate that while these advanced methods strengthen the analytical rigor, they cannot fully compensate for the fundamental constraints of a non-randomized design, and the results should be interpreted as robust associations rather than proven causal effects. This challenge is common in real-world educational research where randomization is often logistically or ethically constrained (41). Finally, the possibility of instructor bias, though statistically adjusted for, cannot be entirely eliminated, as subtle differences in enthusiasm for or promotion of the crossword tool could have influenced student participation and effort.

The generalizability of these findings is subject to certain constraints. This study was conducted within a specific medical terminology course at a single institution, with a particular student demographic. The effectiveness of crossword puzzles may vary across different cultural contexts, education systems, disciplines, and levels of learners’ prior knowledge. Further, the associations observed here relate to short-term test performance; the long-term retention of knowledge facilitated by crossword puzzles remains an open and critical question.

Future research should prioritize several key directions to build upon this work. First, randomized controlled trials (RCTs) are essential to establish causal efficacy and control for unmeasured confounding. Second, studies should investigate the long-term impact of such interventions on knowledge retention over subsequent semesters or years, and their transfer to healthcare management applications. Third, research should explore the differential effectiveness of crossword puzzles across diverse learner subgroups and educational settings to better understand contextual moderators. Fourth, as noted, future work should seek to disentangle the cognitive benefits of the puzzle format from the motivational effects of any associated incentives or gamification elements. Finally, investigating blended learning models that integrate crosswords with complementary strategies, such as SLOTs or spaced repetition systems, could provide a pathway to optimize outcomes for both foundational recall and higher-order conceptual understanding.

Despite its limitations, this study has practical implications for instructional design. The consistent association between crossword use and improved performance, especially in terminology recall, suggests that educators may consider integrating them as a low-cost, supplemental learning tool within a broader pedagogical toolkit. This approach aligns with evidence supporting the use of multimodal and frequent, low-stakes assessments to reinforce learning (42, 43).

However, our findings also suggest that crossword puzzles alone may be insufficient for fostering the deep conceptual understanding required for complex term construction, particularly in complex subjects that require higher-order cognitive skills. Therefore, a blended approach is recommended, combining crossword puzzles with other active, student-centered strategies to cater to varied learning objectives and student needs.

## 5 Conclusion

This study provides evidence of a significant positive association between the use of crossword puzzles and improved test performance in medical education, particularly for term definition and short-answer recall, and most notably among previously lower-performing students. While crosswords appear to enhance specific recall-based competencies, their impact on more complex cognitive tasks may be limited. The integration of complementary instructional strategies, such as student-led tutorials, may help address this gap. Methodologically, the study employed advanced techniques to address confounding in a non-randomized setting, but the design limitations necessitate cautious, associative interpretation of the results. Future research employing randomized designs, investigating long-term retention, and examining blended learning models will be crucial to validate and extend these findings, ultimately helping educators deploy effective, engaging tools to strengthen foundational competencies in healthcare education.

## Data Availability

The raw data supporting the conclusions of this article will be made available by the authors, without undue reservation.

## Footnotes

1
https://www.theteacherscorner.net/
