# Supplementary material for: Improving learning outcomes of medical terminology course through classroom-based gamified crossword puzzle activities
Source: Front Med (Lausanne). 2026 Jan 16;13:1705623. doi: 10.3389/fmed.2026.1705623 (PMC12855051; doi:10.3389/fmed.2026.1705623)
Supplement: Supplementary file 1 [file Supplementary_file_1.docx]

Supplementary Material

# Introduction

The test paper consisted of three sections or parts: Term-Building (T-B), Term-Defining (T-D), and Short Answer Question (SAQ), with a total possible score of 80 marks. In Part A (Term-Building), students were required to construct medical terms based on the descriptions provided. This section contained 20 descriptions, with one mark awarded per correct answer, for a total of 20 marks. Part B (Term-Defining) required students to define specific medical terms, particularly those that were compounded (e.g., myasthenia gravis) or could not be easily defined by word components (e.g., anaemia). This section included 10 terms, with two marks awarded per correct definition, making a total of 20 marks. Part C (Short Answer Questions) consisted of four questions, each with two sub-questions (A and B). Sub-question A required students to answer in a listing format, while sub-question B required a brief explanation. Each question carried 10 marks, contributing to a total of 40 marks. Students were required to answer all questions in English.

# Methods

To evaluate the psychometric properties of the test paper, we analyzed students' answers using both descriptive and inferential statistics. We began by calculating the frequencies and percentages of correct answers, as well as the marks earned for each section of the question paper. Summaries of these descriptive analyses are provided in the appendix of this document.

Next, we applied Item Response Theory (IRT) models for a more in-depth analysis of the data. Given the nature of the responses, we considered dichotomous one-parameter logistic (1PL) and two-parameter logistic (2PL) models for analyzing responses in Parts A and B. For Part C, which uses a polytomous rating scale, we considered the rating scale model (RSM) and the graded response model (GRM). Since the questions in Part C assess two different skills—namely, the ability to list points and the ability to provide brief explanations—we conducted separate GRM and RSM analyses for sub-questions A (items C1a, C2a, C3a, and C4a) and sub-questions B (C1b, C2b, C3b, and C4b).

To select the best models fitting our data, we compared the fit statistics among different Item Response Theory (IRT) models. Given that our dataset is relatively small and our primary goal is prediction accuracy, we focused specifically on Akaike’s Information Criterion (AIC) values to determine which model best fits the parameters. After these initial analyses, we conducted likelihood ratio tests by nesting a simpler IRT model within a more complex one. Based on these analyses, we chose the 2-Parameter Logistic (2PL) model and the Graded Response Model (GRM) to analyze the data further. **Supplementary Table 1** summarizes the fit statistics used for model selection.

| **Supplementary Table 1.** Fit Statistics for Model Selection | | | | | |
| --- | --- | --- | --- | --- | --- |
| IRT Model | Part A | Part B | IRT Model | Part C - a | Part C - b |
| 1-PL |  |  | RSM |  |  |
| AIC | 4038.047 | 2033.021 | AIC | 1757.472 | 2351.912 |
| BIC | 4108.436 | 2069.891 | BIC | 1784.287 | 2385.431 |
| 2-PL |  |  | GRM |  |  |
| AIC | 3995.633 | 1981.159 | AIC | 1732.446 | 2322.895 |
| BIC | 4129.707 | 2048.196 | BIC | 1799.483 | 2416.747 |
| 1-PL vs 2-PL |  |  | RSM vs GRM |  |  |
| LR chi-squared | 80.40, *p* <.001 | 69.86, *p* <.001 | LR chi-squared | 49.03, *p* <.001 | 65.02, *p* <.001 |
| *Abbreviation: IRT = Item Response Theory, 1PL = 1-Parameter Logistic, 2PL = 2-Parameter Logistic, RSM = Rating Scale Model, GRM = Graded Response Model, AIC = Akaike’s information criterion, BIC = Bayesian information criterion* | | | | | |

For the 2PL and GRM models, we calculated both discrimination and difficulty parameters for each item. The discrimination coefficient (a) measures how effectively an item distinguishes between individuals with higher and lower levels of the latent trait. The interpretation of the discrimination level based on the coefficient is as follows: a coefficient of ≤ 0.64 is considered low, 0.65 – 1.34 is moderate, 1.35 – 1.69 is high, and ≥ 1.70 is regarded as very highly discriminating (1,2). Conversely, the difficulty coefficient (b) quantifies how challenging an item is by indicating the level of the latent trait required to have a 50% chance of answering the item correctly. Generally, higher values indicate greater difficulty, meaning that a higher level of ability is needed for a correct response. The levels of difficulty based on estimated parameter values can be interpreted as follows: less than -1 is considered easy, -1 to +1 is moderate difficulty, and more than +1 is classified as high difficulty (1,2). Based on the computed estimates, we plotted and graphically presented the item information functions (IIF), item characteristic curves (ICC)/boundary characteristic curves (CCC), test characteristic curves (TCC)/category characteristic curves (CCC), and the test information function (TIF).

Reliability tests on the assessment items were conducted according to the sections of the question paper. For Parts A and B, internal consistency reliability was evaluated using the Kuder-Richardson reliability test (KR-20). Items in Part C were assessed using Cronbach’s alpha statistics. Additionally, we estimated a more robust statistic within the Item Response Theory (IRT) framework known as the Person Separation Index (PSI). The PSI measures an instrument's ability to differentiate between varying levels of latent traits among test-takers (3, 4). Unlike traditional reliability measures, which rely on raw scores (e.g., Cronbach’s Alpha or KR-20), the PSI is a logit-based measure. This means it utilizes the estimated abilities of each student and accounts for measurement errors related to those estimates. Generally, a PSI value greater than 0.7 is considered acceptable reliability. However, for individual evaluations with significant personal implications, such as diagnostic assessments, PSI values exceeding 0.8 or 0.9 are often preferred (5).

We also conducted inter-rater reliability tests to evaluate the agreement among the raters. In this study, three instructors graded the answer scripts independently. Each instructor initially graded the scripts of their own students, while the other two instructors served as the second and third examiners for this process. Inter-rater reliability was assessed using percentage agreement, Krippendorff’s alpha, and the intraclass correlation coefficient (ICC). Percentage agreement was calculated to provide a straightforward measure of observed concordance across raters. Krippendorff’s alpha was employed as a robust statistic suitable for nominal and ordinal data, which accounts for agreement occurring by chance and accommodates multiple raters (6). The ICC (two-way random effects model, consistency, multiple raters) was applied to evaluate the consistency of assigned marks across raters, as it is the recommended statistic for continuous or interval-level scoring. All analyses were performed using Stata version 18 (StataCorp LLC, College Station, TX, USA).

# Results

## Analyses on Part A Items

This part measures students' ability to construct medical terms based on descriptions provided. This section consisted of 20 items, and each correct identification of a medical term was awarded one mark. The following are examples of the description and answers:

*Instruction:* Construct the medical terms based on the description provided.

1. A situation of excessive swallowing of air while eating or drinking, and is a common cause of gas in the stomach. *(Answer: Aerophagia)*
2. A congenital herniation of brain tissue through a gap in the skull. *(Answer: Encephalocele)*

The results of the 2PL analysis revealed that the discrimination coefficients for all 20 items ranged from 0.90 to 3.26. Specifically, the discrimination coefficients for items 1, 2, and 20 fall between 0.65 and 1.34, indicating a moderate level of discrimination. In contrast, the other items demonstrate a good discrimination index, as their coefficients exceed 1.34. When examining the estimates of the difficulty parameters, almost all items are considered moderately difficult. Only one item, Item A20, has a coefficient greater than 1, which classifies it as a high-difficulty item. **Supplementary Table 2** provides a summary of discrimination and difficulty parameters for all items contained in part A of the question paper.

**Supplementary Figure 1** displays the item information functions (IIFs), which are unimodal and asymmetric. This figure illustrates that each item provides the maximum amount of information at its estimated difficulty parameter. The height of an IIF and the amount of information an item provides around the difficulty parameter are proportional to the item’s estimated discrimination. Based on this figure, items A8 and A14 exhibit the highest discrimination because they have the steepest IIFs, while item A20 has the lowest discrimination, as shown by its shallow slope. **Supplementary Figure 2** compares the item characteristic curves (ICCs) for items with the highest and the lowest discrimination index (A20 vs. A8). The figure shows that the ICC for item A8 has the steepest slope at its estimated difficulty parameter compared to the slopes of the ICCs for item A20. Conversely, the ICC for item A20 displays a more gradual slope, which reflects its low discrimination estimate.

**Supplementary Table 2**. *Discrimination and Difficulty Coefficients for Part A Items*

|  | Discrimination | | | | | Difficulty | | | | |
| --- | --- | --- | --- | --- | --- | --- | --- | --- | --- | --- |
| Item | Coef. | SE | *z* | *p* | 95% CI | Coef. | SE | *z* | *p* | 95% CI |
| A1 | 1.00 | 0.22 | 4.53 | <.001 | 0.56 – 1.43 | 0.95 | 0.23 | 4.14 | <.001 | 0.50 – 1.40 |
| A2 | 1.27 | 0.25 | 5.03 | <.001 | 0.78-1.76 | 0.81 | 0.18 | 4.58 | <.001 | 0.46 – 1.16 |
| A3 | 1.56 | 0.29 | 5.32 | <.001 | 0.99 – 2.14 | 0.71 | 0.15 | 4.80 | <.001 | 0.42 – 1.01 |
| A4 | 1.79 | 0.33 | 5.43 | <.001 | 1.14 – 2.43 | 0.66 | 0.13 | 4.96 | <.001 | 0.40 – 0.92 |
| A5 | 2.10 | 0.38 | 5.48 | <.001 | 1.35 – 2.85 | 0.64 | 0.12 | 5.24 | <.001 | 0.40 – 0.88 |
| A6 | 2.45 | 0.46 | 5.38 | <.001 | 1.56 – 3.35 | 0.61 | 0.11 | 5.39 | <.001 | 0.39 – 0.83 |
| A7 | 2.97 | 0.56 | 5.27 | <.001 | 1.87 – 4.08 | 0.58 | 0.10 | 5.59 | <.001 | 0.38 – 0.78 |
| A8 | 3.53 | 0.71 | 4.95 | <.001 | 2.14 – 4.93 | 0.56 | 0.10 | 5.74 | <.001 | 0.37 – 0.75 |
| A9 | 2.62 | 0.48 | 5.44 | <.001 | 1.67 – 3.56 | 0.60 | 0.11 | 5.47 | <.001 | 0.38 – 0.81 |
| A10 | 2.56 | 0.45 | 5.64 | <.001 | 1.67 – 3.46 | 0.60 | 0.11 | 5.44 | <.001 | 0.39 – 0.81 |
| A11 | 2.94 | 0.53 | 5.52 | <.001 | 1.90 – 3.99 | 0.58 | 0.10 | 5.59 | <.001 | 0.38 – 0.78 |
| A12 | 2.95 | 0.55 | 5.34 | <.001 | 1.87 – 4.04 | 0.58 | 0.10 | 5.60 | <.001 | 0.38 – 0.78 |
| A13 | 3.23 | 0.65 | 4.98 | <.001 | 1.96 – 4.51 | 0.57 | 0.10 | 5.66 | <.001 | 0.37 – 0.77 |
| A14 | 3.26 | 0.66 | 4.91 | <.001 | 1.96 – 4.57 | 0.58 | 0.10 | 5.78 | <.001 | 0.39 – 0.78 |
| A15 | 2.46 | 0.47 | 5.23 | <.001 | 1.54 – 3.38 | 0.62 | 0.11 | 5.50 | <.001 | 0.40 – 0.85 |
| A16 | 2.09 | 0.38 | 5.45 | <.001 | 1.34 – 2.85 | 0.64 | 0.12 | 5.25 | <.001 | 0.40 – 0.88 |
| A17 | 1.92 | 0.35 | 5.49 | <.001 | 1.24 – 2.61 | 0.67 | 0.13 | 5.21 | <.001 | 0.42 – 0.93 |
| A18 | 1.55 | 0.29 | 5.30 | <.001 | 0.98 – 2.14 | 0.74 | 0.15 | 4.88 | <.001 | 0.44 – 1.03 |
| A19 | 1.59 | 0.30 | 5.40 | <.001 | 1.01 – 2.17 | 0.74 | 0.15 | 5.01 | <.001 | 0.45 – 1.03 |
| A20 | 0.90 | 0.21 | 4.28 | <.001 | 0.49 – 1.32 | 1.04 | 0.26 | 4.00 | <.001 | 0.53 – 1.56 |

**Supplementary Figure 1.**  *Item Information Functions for Part A (Term-Building) Items.*

Supplementary Figure 2. Item Characteristic Curve for Part A Item (Term-Building).

The test characteristic curve (TCC), shown in **Supplementary Figure 3**, indicates that expected scores range from 0 to 20. According to the 95% critical values from the standard normal distribution, above-average students are expected to score four marks or higher. Additionally, the test information function (TIF), presented in **Supplementary Figure 4**, assesses how effectively the test differentiates between examinees at various levels of a latent trait. In simpler terms, the TIF plot evaluates the instrument's ability to estimate individual locations on the trait continuum. The results reveal that maximum information for individuals is located at approximately *θ* = 0.60 (IIF = 28.9, *SE* = 0.19). As we move away from this point in either direction, the standard error of the TIF increases, meaning the instrument provides less reliable information about *θ*.

Supplementary Figure 3. Test Characteristic Curve for Part A Items (Term-Building).

Supplementary Figure 4. Test Information Function for Part A Items (Term-Building).

## Analyses on Part B Items

Questions in this part measured students' ability to define medical terms properly. This section consisted of 10 items, and each correctly defined medical term would be awarded two marks. The following are examples of terms and answers:

*Instruction:* Provide an appropriate definition for the terms listed below:

1. Ptosis

*(Answer: The drooping of the upper eyelid that is usually due to muscle weakness or paralysis)*

1. Celiac disease

*(Answer: An autoimmune disorder, which is characterized by several reactions to ingesting gluten, which then damages the villi of the small intestine and can cause malabsorption.)*

The results of the 2PL analysis revealed that the discrimination coefficients for all 10 items ranged from 0.84 to 5.83. Specifically, the discrimination coefficients for items B1, B2, and B10 fall between 0.65 and 1.34, indicating a moderate level of discrimination. In contrast, the other items demonstrate a good discrimination index, as their coefficients exceed 1.34. When examining the estimates of the difficulty parameters, all items are considered moderately difficult as their coefficients range from -1 to +1. **Supplementary Table 3** summarizes the results of 2PL analyses on Part B items.

**Supplementary Table 3.** *Discrimination and Difficulty Coefficients for Part B Items*

|  | Discrimination | | | | | Difficulty | | | | |
| --- | --- | --- | --- | --- | --- | --- | --- | --- | --- | --- |
| Item | Coef. | SE | z | *p* | 95% CI | Coef. | SE | z | *p* | 95% CI |
| B1 | 0.84 | 0.21 | 3.92 | <.001 | 0.42 – 1.26 | 1.04 | 0.28 | 3.70 | <.001 | 0.49 – 1.60 |
| B2 | 1.31 | 0.27 | 4.87 | <.001 | 0.78 – 1.84 | 0.84 | 0.18 | 4.82 | <.001 | 0.50 – 1.19 |
| B3 | 2.48 | 0.46 | 5.33 | <.001 | 1.57 – 3.39 | 0.67 | 0.11 | 6.05 | <.001 | 0.45 – 0.88 |
| B4 | 2.70 | 0.52 | 5.25 | <.001 | 1.69 – 3.71 | 0.67 | 0.11 | 6.28 | <.001 | 0.46 – 0.88 |
| B5 | 3.51 | 0.72 | 4.86 | <.001 | 2.10 – 4.94 | 0.67 | 0.10 | 6.86 | <.001 | 0.48 – 0.86 |
| B6 | 5.83 | 1.67 | 3.49 | <.001 | 2.56 – 9.10 | 0.65 | 0.09 | 7.42 | <.001 | 0.48 – 0.83 |
| B7 | 2.90 | 0.57 | 5.11 | <.001 | 1.79 – 4.02 | 0.72 | 0.11 | 6.80 | <.001 | 0.51 – 0.93 |
| B8 | 2.97 | 0.60 | 4.95 | <.001 | 1.79 – 4.14 | 0.79 | 0.11 | 7.23 | <.001 | 0.57 – 1.00 |
| B9 | 2.14 | 0.42 | 5.16 | <.001 | 1.33 – 2.95 | 0.80 | 0.12 | 6.34 | <.001 | 0.55 – 1.05 |
| B10 | 1.20 | 0.26 | 4.54 | <.001 | 0.68 – 1.71 | 1.08 | 0.22 | 4.96 | <.001 | 0.66 – 1.50 |

**Supplementary Figure 5** displays the item information functions (IIFs), which are unimodal and asymmetric. This figure illustrates that each item provides the maximum amount of information at its estimated difficulty parameter. The height of an IIF and the amount of information an item provides around the difficulty parameter are proportional to the item’s estimated discrimination. Based on this figure, items B6 and B5 exhibit the highest discrimination because they have the steepest IIFs, while item B1 has the lowest discrimination, as shown by its shallow slope.

**Supplementary Figure 6** compares the item characteristic curves (ICCs) for two items with the highest and the lowest discrimination index (B6 vs. B1). The figure shows that the ICC for item B6 shows the steepest slope at its estimated difficulty parameter compared to the slopes of the ICCs of other items. Conversely, the ICC for item B1 displays a more gradual slope, which reflects its low discrimination estimate.

Supplementary Figure 5. Item Information Functions for Part B Items (Term-Building).

Supplementary Figure 6. Item Characteristic Curves for Part B Items (Term-Defining).

The test characteristic curve (TCC), shown in **Supplementary Figure 7**, indicates that expected scores range from 0 to 10. According to the 95% critical values for the standard normal distribution, above-average students are expected to correctly define two or more terms (scoring ≥ 4 marks). Additionally, the test information function (TIF), presented in **Supplementary Figure 8**, assesses how effectively the test differentiates between examinees at various levels of a latent trait. In simpler terms, the TIF plot evaluates the instrument's ability to estimate individual locations on the trait continuum. The results reveal that maximum information for individuals is located at approximately *θ* = 0.68 (IIF = 22.2, *SE* = 0.21). As we move away from this point in either direction, the standard error of the TIF increases, meaning the instrument provides less reliable information about *θ*.

Supplementary Figure 7. Test Characteristic Curve for Part B Items (Term-Defining)

Supplementary Figure 8. Test Information Function for Part B Items (Term-Defining)

## Analyses on Part C Items

Part C questions were intended to measure students’ ability to write short answers to the questions provided. This part consists of four questions, and each question has sub-questions A and B. Sub-question A asks the students to provide the answer in a list format, whereas sub-question B asks students to answer the question with a brief explanation. As the questions in Part C measure two different sets of abilities, we performed separate GRM analyses for sub-questions A (items C1a, C2a, C3a, and C4a) and sub-questions B (C1b, C2b, C3b, and C4b). Examples of the questions are as follows:

Question 1 (a)

*List four functions of the hypothalamus. (4 marks)*

Question 1 (b)

*Briefly explain any three methods of assisted reproduction (6 marks)*

*Analyses on Sub-questions C1a, C2a, C3a and C4a.*

The results of the GRM analysis revealed that the discrimination coefficients for all four items ranged from 1.15 to 3.10. The discrimination coefficient for item 1a falls between 0.65 and 1.34, indicating a moderate level of discrimination. In contrast, the other items demonstrate a good discrimination index, as their coefficients exceed 1.34.

The difficulty parameter estimates of GRM are based on four thresholds, which represent the mark earned. For items C1a and C4a, the difficulty parameters suggest that achieving 1 or 2 marks, as opposed to not getting any points, was relatively easy compared to obtaining higher scores. For items C2a and C3a, on the other hand, getting 1 point, as opposed to not getting any points, was easier than getting more marks for listing the points. **Supplementary Table 4** summarizes the discrimination and difficulty parameters for part C items (C1a, C2a, C3a, and C4a).

**Supplementary Table 4.** *Discrimination and Difficulty Coefficients for Part C(a) Items*

|  | Discrimination | | | | | Difficulty | | | |
| --- | --- | --- | --- | --- | --- | --- | --- | --- | --- |
| Item | Coef. | SE | z | *p* | 95% CI | Score | Coef. | SE | 95% CI |
| 1a | 1.15 | 0.23 | 4.91 | <.001 | 0.69 – 1.61 | ≥ 1 | -3.25 | 0.59 | -4.40, -2.10 |
|  |  |  |  |  |  | ≥ 2 | -2.61 | 0.45 | --3.50, -1.71 |
|  |  |  |  |  |  | ≥ 3 | -1.73 | 0.31 | -2.33, -1.13 |
|  |  |  |  |  |  | 4 | -0.51 | 0.16 | -0.83, -0.19 |
| 2a | 3.10 | 0.86 | 3.61 | <.001 | 1.42 – 4.78 | ≥ 1 | -2.02 | 0.24 | -2.48, -1.54 |
|  |  |  |  |  |  | ≥ 2 | -1.69 | 0.20 | -2.07, -1.30 |
|  |  |  |  |  |  | ≥ 3 | -1.18 | 0.15 | -1.47, -0.89 |
|  |  |  |  |  |  | 4 | -0.43 | 0.10 | -0.63, -0.23 |
| 3a | 2.10 | 0.41 | 5.07 | <.001 | 1.29 – 2.90 | ≥ 1 | -2.42 | 0.32 | -3.05, -1.78 |
|  |  |  |  |  |  | ≥ 2 | -1.83 | 0.23 | -2.28, -1.39 |
|  |  |  |  |  |  | ≥ 3 | -1.41 | 0.18 | -1.78, -1.05 |
|  |  |  |  |  |  | 4 | -0.29 | 0.11 | -0.51, -0.70 |
| 4a | 1.45 | 0.27 | 5.38 | <.001 | 0.92 – 1.98 | ≥ 1 | -2.79 | 0.43 | -3.64, -1.95 |
|  |  |  |  |  |  | ≥ 2 | -2.12 | 0.31 | -2.73, -1.52 |
|  |  |  |  |  |  | ≥ 3 | -1.62 | 0.24 | -2.09, -1.15 |
|  |  |  |  |  |  | 4 | -0.34 | 0.13 | -0.61, -0.08 |

**Supplementary Figure 9** displays the item information functions (IIFs) for all items. This figure illustrates that each item provides the maximum amount of information at its estimated difficulty parameter. The height of an IIF and the amount of information an item provides around the difficulty parameter are proportional to the item’s estimated discrimination. Based on this figure, items C2a and C3a exhibit the highest discrimination because they have the steepest IIFs, while item C1a has the lowest discrimination, as shown by its shallow slope. **Supplementary Figure 10** presents the boundary characteristic curves (BCCs) for all sub-question A items (C1a, C2a, C3a, and C4a). Because the estimated discrimination parameters for sub-questions C1a and C4a are relatively smaller than sub-questions C2a and C3a, the curves have relatively flat slopes.

Supplementary Figure 9. Item Information Functions for Part C Items (Short Answer - Listing)

**
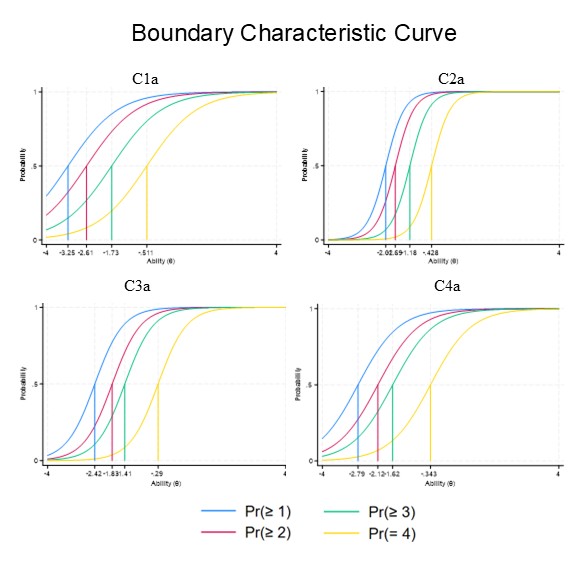
**

Supplementary Figure 10. Boundary Characteristic Curves for Part C items (Short Answer - Listing)

The category characteristic curves (CCC), shown in **Supplementary Figure 11**, show category probabilities, Pr(Y = k), as a function of *θ.* The graph shows that students with the latent trait level below approximately *θ* = -1.35 are most likely to be able to list one correct point for each item. The possibility of students being able to list all four points correctly increases when the latent trait level is between *θ* = -1.35 and *θ* = 1.55.

**
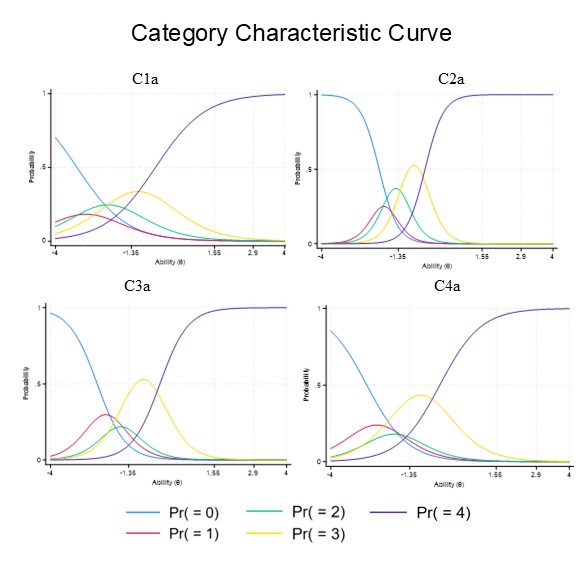
**

Supplementary Figure 11. Category Characteristic Curve for Part C Items (Short Answer - Listing)

The test characteristic curve, as presented in **Supplementary Figure 12**, indicates that expected scores range from 0 to 16. According to the 95% critical values from the standard normal distribution, above-average students are expected to be able to list six points or more correctly. Additionally, the test information function (TIF), presented in **Supplementary Figure 13**, assesses how effectively the test differentiates between examinees at various levels of a latent trait. In simpler terms, the TIF plot evaluates the instrument's ability to estimate individual locations on the trait continuum. The results reveal that maximum information is available for individuals situated at approximately *θ* = -1.51 (IIF = 31.54, SE = 0.18). As we move away from this point in either direction, the standard error of the TIF increases, meaning the instrument provides less reliable information about *θ*.

Supplementary Figure 12. Test Characteristic Curve for Part C Items (Short Answer - Listing)

Supplementary Figure 13. Test Information Function for Part C Items (Short Answer - Listing)

*Analyses on Sub-questions C1b, C2b, C3b, and C4b.*

The results of the GRM analysis revealed that the discrimination coefficients for all four items ranged from 2.35 to 4.19, suggesting all items have a good discrimination index.

The difficulty parameter estimates of GRM are based on size thresholds, which represent the mark earned. For items C1b and C4b, the difficulty parameters suggest that achieving 1 or 2 points was relatively easy compared to higher scores. For items C2b and C3b, on the other hand, getting 1 point is easier than getting more points. Although it is easy to get marks in this section, getting more than five marks is statistically difficult as the coefficient is greater than 1. **Supplementary Table 5** summarizes the discrimination and difficulty parameters for part Cb items.

| **Supplementary Table 5.** *Discrimination and Difficulty Coefficients for Part C(b) Items* | | | | | | | | | |
| --- | --- | --- | --- | --- | --- | --- | --- | --- | --- |
|  | Discrimination | | | | | Difficulty | | | |
| Item | Coef. | SE | z | *p* | 95% CI | Score | Coef. | SE | 95% CI |
| C1b | 2.35 | 0.30 | 7.81 | <.001 | 1.76 – 2.94 | ≥ 1 | -0.29 | 0.30 | -0.50, -0.08 |
|  |  |  |  |  |  | ≥ 2 | -0.16 | 0.11 | -0.36 – 0.04 |
|  |  |  |  |  |  | ≥ 3 | 0.02 | 0.10 | -0.18 – 0.22 |
|  |  |  |  |  |  | ≥ 4 | 0.73 | 0.12 | 0.05 – 0.96 |
|  |  |  |  |  |  | ≥ 5 | 1.17 | 0.14 | 0.90 – 1.45 |
|  |  |  |  |  |  | 6 | 1.68 | 0.18 | 1.32 – 2.03 |
| C2b | 4.19 | 0.66 | 6.31 | <.001 | 2.89 – 5.49 | ≥ 1 | -0.11 | 0.09 | -0.28 – 0.06 |
|  |  |  |  |  |  | ≥ 2 | 0.03 | 0.09 | -0.14 – 0.20 |
|  |  |  |  |  |  | ≥ 3 | 0.24 | 0.09 | 0.07 – 0.41 |
|  |  |  |  |  |  | ≥ 4 | 0.77 | 0.77 | 0.57 – 0.97 |
|  |  |  |  |  |  | ≥ 5 | 1.14 | 1.14 | 0.91 – 1.37 |
|  |  |  |  |  |  | 6 | 1.51 | 1.51 | 1.22 – 1.79 |
| C3b | 3.85 | 0.56 | 6.83 | <.001 | 2.74 – 4.95 | ≥ 1 | -0.14 | 0.09 | -0.31 – 0.04 |
|  |  |  |  |  |  | ≥ 2 | 0.05 | 0.09 | -0.12 – 0.22 |
|  |  |  |  |  |  | ≥ 3 | 0.29 | 0.09 | 0.12 – 0.47 |
|  |  |  |  |  |  | ≥ 4 | 0.82 | 0.10 | 0.61 – 1.02 |
|  |  |  |  |  |  | ≥ 5 | 1.14 | 0.12 | 0.90 – 1.37 |
|  |  |  |  |  |  | 6 | 1.66 | 0.16 | 1.35 – 1.97 |
| C4b | 3.37 | 0.49 | 6.96 | <.001 | 2.42 – 4.32 | ≥ 1 | -0.24 | 0.09 | -0.42, -0.05 |
|  |  |  |  |  |  | ≥ 2 | -0.07 | 0.09 | -0.24 – 0.11 |
|  |  |  |  |  |  | ≥ 3 | 0.16 | 0.09 | -0.02 – 0.34 |
|  |  |  |  |  |  | ≥ 4 | 1.19 | 0.13 | 0.94 – 1.44 |
|  |  |  |  |  |  | ≥ 5 | 1.48 | 0.15 | 1.19 – 1.77 |
|  |  |  |  |  |  | 6 | 2.01 | 0.20 | 1.62 – 2.41 |

**Supplementary Figure 14.** displays the item information functions (IIFs) for all items. This figure illustrates that each item provides the maximum amount of information at its estimated difficulty parameter. The height of an IIF and the amount of information an item provides around the difficulty parameter are proportional to the item’s estimated discrimination. Based on this figure, items C2b and C3b exhibit the highest discrimination because they have the steepest IIFs, while item C1b has the lowest discrimination, as shown by its shallow slope. Supplementary Figure 15 presents the boundary characteristic curves (ICCs) for all sub-question B items (C1b, C2b, C3b, and C4b). Because the estimated discrimination parameters for sub-questions C1b and C4b are relatively smaller than sub-questions C2b and C3b, the curves have relatively flat slopes.

Supplementary Figure 14. Item Information Functions for Part C Items (Short Answer - Explaining)

**
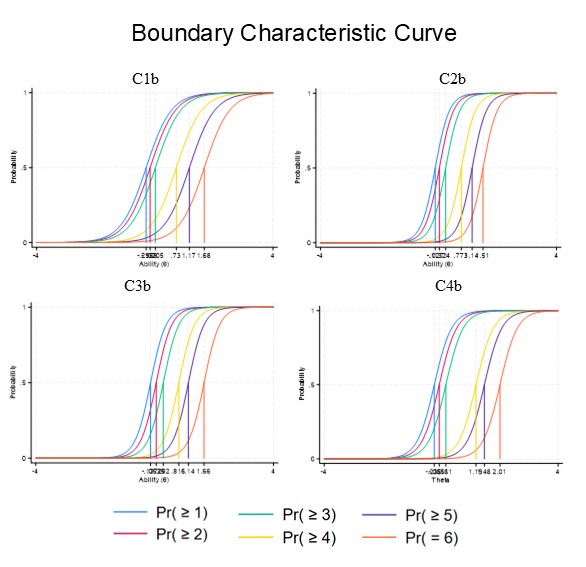
**

Supplementary Figure 15. Boundary Characteristic Curve for Part C Items (Short Answer - Explaining)

The category characteristic curves (CCC), shown in **Supplementary Figure 16**, show category probabilities, Pr(Y = k), as a function of *θ.* The graph shows that students with the latent trait level below approximately *θ* = -1.35 are most likely unable to get any mark for all items. The possibility of students being able get marks for their explanation increases when the latent trait level is between *θ* = -1.35 and *θ* = 1.55. Statistically, only those with latent trait levels *θ* ≥ 1.55 would be able to get full marks on any questions from this part.

**
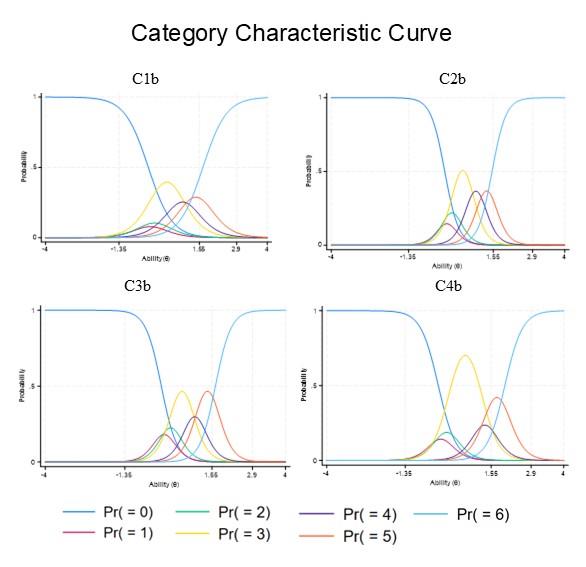
**

Supplementary Figure 16. Category Characteristic Curve for Part C Items (Short Answer - Explaining)

The test characteristic curve, as presented in **Supplementary Figure 17**, indicates that expected scores range from 0 to 24. According to the 95% critical values from the standard normal distribution, above-average students are expected to be able to list six points or more correctly. Additionally, the test information function (TIF), presented in **Supplementary Figure 18**, assesses how effectively the test differentiates between examinees at various levels of a latent trait. In simpler terms, the TIF plot evaluates the instrument's ability to estimate individual locations on the trait continuum. The results reveal that maximum information is available for individuals situated at approximately *θ* = 1.22 (IIF = 15.91, SE = 0.25). As we move away from this point in either direction, the standard error of the TIF increases, meaning the instrument provides less reliable information about *θ*.

Supplementary Figure 17. Test Characteristic Curve for Part C Items (Short Answer - Explaining)

Supplementary Figure 18. Test Information Function for Part C (Short Answer - Explaining)

## Reliability Analysis

Due to the differences in the types of item responses, we performed reliability analyses using three primary methods. For Parts A and B, we utilized the Kuder-Richardson formula (KR-20), while for Part C, we applied Cronbach’s Alpha to evaluate reliability (7,8). Additionally, we calculated the Person Separation Index (PSI) to assess internal consistency of the items within the Item Response Theory (IRT) framework.

The results of these analyses indicated that the items in Part A demonstrated excellent reliability (α = 0.92). In contrast, the items in Parts B (α = 0.86) and C (sub-question b: α = 0.88) exhibited good reliability (7). Items in Part C, specifically sub-questions A, along with the combined items (containing sub-questions A and B), showed acceptable reliability overall (8). The calculated PSI values further suggest that the items in each section and subsection of the question paper achieved acceptable reliability. **Supplementary Table 7** provides a summary of the results from the reliability analyses conducted.

**Supplementary Table 7.** Reliability of the final test paper according to the sections.

| Section | No. of Items | alpha | PSI^b^ |
| --- | --- | --- | --- |
| A | 20 | 0.92^a^ | 0.88 |
| B | 10 | 0.86^a^ | 0.81 |
| Ca | 4 | 0.70 | 0.72 |
| Cb | 4 | 0.88 | 0.86 |
| Ca + Cb | 8 | 0.79 | 0.85 |
| *Abbreviation: PSI = Person Separation Index*  *Note: the reliability coefficient is based on the Kuder-Richardson formula*  *PSI values were calculated based on the IRT Rating Scale Model within the Rasch Measurement Theory framework.* | | | |

## Interrater Agreement

A total of 211 answer scripts from the medical terminology final test were independently graded by three instructors. Inter-rater reliability was examined for each section of the paper (Part A, Part B, and Part C), with Part C further analyzed by sub-sections (A: listing, and B: explaining).

For Part A and Part B, the level of agreement was consistently high, with percentage agreement and Krippendorff’s alpha both at 0.98 (SEs = 0.005–0.007, *p* < .001), and ICC values of 0.99 (*F* (210.0, 420.0) = 16,487.06 and 4,173.14, respectively, *p* < .001). Part C also demonstrated strong reliability. Sub-question A (listing) showed percentage agreement of 0.97, Krippendorff’s alpha of 0.96 (SEs = 0.009–0.011, *p* < .001), and ICC of 0.99 (*F* = 1,925.79, *p* < .001), while sub-question B (explaining) yielded nearly identical results (percentage agreement = 0.97, alpha = 0.97, ICC = 0.99, *F* = 11,579.70, *p* < .001). When combined, Part C maintained high reliability, with percentage agreement and Krippendorff’s alpha at 0.94 (SEs = 0.012–0.013, *p* < .001) and ICC at 0.99 (*F* = 18,005.10, *p* < .001).

Analysis of the total score across all sections confirmed a high degree of consistency among raters, with percentage agreement and Krippendorff’s alpha both at 0.92 (SE = 0.014, *p* < .001) and ICC at 0.99 (*F* = 13,540.40, *p* < .001). Collectively, these results demonstrate robust inter-rater reliability, providing strong evidence for the validity of the grading process employed in this examination. **Supplementary** **Table 8** provides a summary of inter-rater reliability statistics obtained from the analyses.

**Supplementary Table 8.** *Summary of results of the Interrater agreement analysis based on three raters*

|  | Coef. | *SE* | *t* | *p* | 95% CI |
| --- | --- | --- | --- | --- | --- |
| **Part A** |  |  |  |  |  |
| % agreement | 0.98 | 0.005 | 157.38 | <.001 | 0.98 – 0.99 |
| Krippendorff’s Alpha | 0.98 | 0.006 | 144.84 | <.001 | 0.97 – 0.99 |
| Interclass correlation (ICC) | 0.99 | *F* (210.0, 420.0) | = 16487.06 | <.001 | 0.99 – 0.99 |
| **Part B** |  |  |  |  |  |
| % agreement | 0.98 | 0.006 | 157.38 | <.001 | 0.97 – 0.99 |
| Krippendorff’s Alpha | 0.98 | 0.007 | 130.50 | <.001 | 0.96 – 0.99 |
| Interclass correlation (ICC) | 0.99 | *F* (210.0, 420.0) | = 4173.14 | <.001 | 0.99 – 0.99 |
| **Part C (all)** |  |  |  |  |  |
| % agreement | 0.94 | 0.012 | 77.97 | <.001 | 0.92 – 0.97 |
| Krippendorff’s Alpha | 0.94 | 0.013 | 74.86 | <.001 | 0.92 – 0.97 |
| Interclass correlation (ICC) | 0.99 | *F* (210.0, 420.0) | = 8005.10 | <.001 | 0.99 – 0.99 |
| 1. Part C (Listing) |  |  |  |  |  |
| % agreement | 0.97 | 0.009 | 104.51 | <.001 | 0.95 – 0.98 |
| Krippendorff’s Alpha | 0.96 | 0.011 | 84.30 | <.001 | 0.94 – 0.98 |
| Interclass correlation (ICC) | 0.99 | *F* (210.0, 420.0) | = 1925.79 | <.001 | 0.99 – 0.99 |
| 1. Part C (Explaining) |  |  |  |  |  |
| % agreement | 0.97 | 0.008 | 110.94 | <.001 | 0.95 – 0.99 |
| Krippendorff’s Alpha | 0.97 | 0.009 | 100.94 | <.001 | 0.95 – 0.99 |
| Interclass correlation (ICC) | 0.99 | *F* (210.0, 420.0) | = 11579.70 | <.001 | 0.99 – 0.99 |
| **Total Score** |  |  |  |  |  |
| % agreement | 0.92 | 0.144 | 63.27 | <.001 | 0.89 – 0.95 |
| Krippendorff’s Alpha | 0.92 | 0.014 | 61.85 | <.001 | 0.89 – 0.95 |
| Interclass correlation (ICC) | 0.99 | *F* (210.0, 420.0) | = 13540.40 | <.001 | 0.99 – 0.99 |

# Conclusion

The psychometric assessment of the medical terminology final test question paper demonstrated generally acceptable properties across multiple statistical evaluations. Analyses using both the 2-parameter logistic (2PL) model and the Graded Response Model (GRM) indicated that items in Parts A and B possessed adequate discrimination parameters, ranging from moderate to high, and reflected a balanced level of difficulty, with no items being excessively easy or overly challenging. The test characteristic curves (TCCs) further confirmed that above-average students could reasonably be expected to achieve a satisfactory level of performance across different task types, including constructing, defining, and explaining medical terms. Reliability analyses, conducted through KR-20, Cronbach’s alpha, and the Person Separation Index (PSI), consistently supported the internal consistency of the test items. Interrater reliability, measured using percent agreement, Krippendorff’s alpha, and intraclass correlations, demonstrated a high level of agreement among raters, thereby supporting the validity of the scoring process.

Nonetheless, certain limitations were observed in the test information function (TIF) and item information function (IIF) analyses. Specifically, the TIF plots, although demonstrating strong discrimination through tall peaks, were not optimally centered at the zero-ability level, particularly for Part C listing questions. Moreover, a subset of items displayed relatively shallow slopes in their IIF plots, suggesting weaker discriminative power compared with other items. While such imperfections are common and rarely avoidable in practical test development, the insights gained from these analyses provide a valuable basis for refining future iterations of the question paper, ensuring continuous improvement in assessment design for the medical terminology course.

# Reference

1. Baker FB. The basics of item response theory (2nd ed.). College Park, MD: ERIC Clearinghouse on Assessment and Evaluation (2001). http://echo.edres.org:8080/irt/baker/ [Accessed March 14, 2025].
2. Baker FB, Kim S-H. (Eds.) Item Response Theory: Parameter Estimation Techniques, Second Edition (2nd ed.). CRC Press (2004). doi: 10.1201/9781482276725
3. Wright B, Stone M. Measurement essentials. Wide Range, Inc. (1999)
4. Andrich D, Marais I. A course in Rasch Measurement Theory: Measuring in the Education, Social, and Health Sciences. Springer (2019)
5. Hardouin JB, Dubuy Y, Blanchin M, Ducarroz S. The Stata module -pcm- for Rasch Measurement Theory -An application to assess Health Literacy determinants for residents of homeless shelters. Hal Open Science [Preprint] (2024): 1-25. Available at: https://hal.science/hal-04812388v1/document
6. Krippendorff K. Estimating the reliability, systematic error and random error of interval data. *Educ* *Psychol Meas* (1970) 30: 61-70. doi: 10.1177/001316447003000105
7. Wombacher K. Reliability, Kuder-Richardson Formula” In The Sage Encyclopedia of Communication Research Methods, SAGE Publications, Inc (2017). 1418-20 pp. doi: 10.4135/9781483381411.n493
8. Cronbach LJ. Coefficient alpha and the internal structure of tests. *Psychometrika* (1951) 16(3): 297-334. doi: 10.1007/BF02310555

# Appendix

**Supplementary Table 9.** *Descriptive statistics of correct answers for part A questions*

| No. of correct answers | All (*n* = 211) | | Instructor A  (*n* = 90) | | Instructor B  (*n* =49) | | Instructor C  (*n* = 72) | |
| --- | --- | --- | --- | --- | --- | --- | --- | --- |
|  | Freq. | % | Freq | % | Freq. | % | Freq | % |
| 0 | 39 | 18.5 | 14 | 15.6 | 11 | 22.5 | 14 | 19.4 |
| 1 | 23 | 10.9 | 6 | 6.7 | 12 | 24.5 | 5 | 6.9 |
| 2 | 16 | 7.58 | 7 | 7.8 | 1 | 2.0 | 8 | 11.1 |
| 3 | 19 | 9.0 | 5 | 5.6 | 4 | 8.2 | 10 | 13.9 |
| 4 | 9 | 4.3 | 2 | 2.2 | 4 | 8.2 | 3 | 4.2 |
| 5 | 13 | 6.2 | 4 | 4.4 | 3 | 6.1 | 6 | 8.3 |
| 6 | 6 | 2.8 | 2 | 2.2 | 1 | 2.0 | 3 | 4.2 |
| 7 | 5 | 2.4 | 1 | 1.1 | 1 | 2.0 | 3 | 4.2 |
| 8 | 13 | 6.2 | 8 | 8.9 | 3 | 6.1 | 2 | 2.8 |
| 9 | 8 | 3.8 | 1 | 1.1 | 3 | 6.1 | 4 | 5.6 |
| 10 | 10 | 4.7 | 6 | 6.7 | 1 | 2.0 | 3 | 4.2 |
| 11 | 4 | 1.9 | 2 | 2.2 | 1 | 2.0 | 1 | 1.4 |
| 12 | 6 | 2.8 | 5 | 5.6 | 0 | 0.0 | 1 | 1.4 |
| 13 | 6 | 2.8 | 1 | 1.1 | 2 | 4.1 | 3 | 4.2 |
| 14 | 7 | 3.3 | 5 | 5.6 | 1 | 2.0 | 1 | 1.4 |
| 15 | 5 | 2.4 | 4 | 4.4 | 0 | 0.0 | 1 | 1.4 |
| 16 | 7 | 3.3 | 4 | 4.4 | 0 | 0.0 | 3 | 4.2 |
| 17 | 4 | 1.9 | 4 | 4.4 | 0 | 0.0 | 0 | 0.0 |
| 18 | 3 | 1.4 | 3 | 3.3 | 0 | 0.0 | 0 | 0.0 |
| 19 | 6 | 2.8 | 4 | 4.4 | 1 | 2.0 | 1 | 1.4 |
| 20 | 2 | 1.0 | 2 | 2.2 | 0 | 0.0 | 0 | 0.0 |

**Supplementary Table 10.** *Descriptive statistics of correct answers for part A questions*

| No. of correct answers | All (*n* = 211) | | Instructor A  (*n* = 90) | | Instructor B  (*n* =49) | | Instructor C  (*n* = 72) | |
| --- | --- | --- | --- | --- | --- | --- | --- | --- |
|  | Freq. | % | Freq | % | Freq. | % | Freq | % |
| 0 | 68 | 32.2 | 23 | 25.6 | 16 | 23.7 | 29 | 40.3 |
| 1 | 31 | 14.7 | 6 | 6.7 | 13 | 26.5 | 12 | 16.7 |
| 2 | 34 | 16.1 | 10 | 11.1 | 10 | 20.4 | 14 | 19.4 |
| 3 | 3 | 1.4 | 1 | 1.1 | 1 | 2.0 | 1 | 1.4 |
| 4 | 14 | 6.6 | 9 | 10.0 | 2 | 4.1 | 3 | 4.2 |
| 5 | 12 | 5.7 | 4 | 4.4 | 1 | 2.0 | 7 | 9.7 |
| 6 | 15 | 7.1 | 12 | 13.3 | 3 | 6.1 | 0 | 0.0 |
| 7 | 13 | .2 | 7 | 7.8 | 2 | 4.1 | 4 | 5.6 |
| 8 | 12 | 5.7 | 10 | 11.1 | 1 | 2.0 | 1 | 1.4 |
| 9 | 5 | 2.4 | 4 | 4.4 | 0 | 0.0 | 1 | 1.4 |
| 10 | 4 | 1.9 | 4 | 4.4 | 0 | 0.0 | 0 | 0.0 |
|  | | | | | | | | |

**Supplementary Table 11.** *Descriptive statistics of mark distribution for part c(a) questions*

| Mark earned | All (*n* = 211) | | Instructor A  (*n* = 90) | | Instructor B  (*n* =49) | | Instructor C  (*n* = 72) | |
| --- | --- | --- | --- | --- | --- | --- | --- | --- |
|  | Freq. | % | Freq | % | Freq. | % | Freq | % |
| 0 | 0 | 0.0 | 0 | 0.0 | 0 | 0.0 | 0 | 0.0 |
| 1 | 0 | 0.0 | 0 | 0.0 | 0 | 0.0 | 0 | 0.0 |
| 2 | 1 | 0.5 | 0 | 0.0 | 0 | 0.0 | 1 | 1.4 |
| 3 | 0 | 0.0 | 0 | 0.0 | 0 | 0.0 | 0 | 0.0 |
| 4 | 3 | 1.4 | 0 | 0.0 | 1 | 2.0 | 2 | 2.8 |
| 5 | 3 | 1.4 | 2 | 2.2 | 0 | 0.0 | 1 | 1.4 |
| 6 | 3 | 1.4 | 2 | 2.2 | 1 | 2.0 | 0 | 0.0 |
| 7 | 2 | 1.0 | 0 | 0.0 | 1 | 2.0 | 1 | 1.4 |
| 8 | 10 | 4.5 | 7 | 7.8 | 2 | 4.1 | 1 | 1.4 |
| 9 | 4 | 1.9 | 0 | 0.0 | 1 | 2.0 | 3 | 4.2 |
| 10 | 5 | 2.4 | 2 | 2.2 | 1 | 2.0 | 2 | 2.8 |
| 11 | 3 | 1.4 | 2 | 2.2 | 0 | 0.0 | 1 | 1.4 |
| 12 | 44 | 20.9 | 20 | 22.2 | 8 | 16.3 | 16 | 22.2 |
| 13 | 12 | 5.7 | 7 | 7.8 | 3 | 6.1 | 2 | 2.8 |
| 14 | 9 | 4.3 | 8 | 8.9 | 1 | 2.0 | 0 | 0.0 |
| 15 | 52 | 24.6 | 18 | 20.0 | 14 | 28.6 | 20 | 278 |
| 16 | 60 | 28.4 | 22 | 24.4 | 16 | 32.7 | 22 | 30.6 |
| . | | | | | | | | |

**Supplementary Table 12.** *Descriptive statistics of mark distribution for part c(b) questions*

| Mark earned | All (*n* = 211) | | Instructor A  (*n* = 90) | | Instructor B  (*n* =49) | | Instructor C  (*n* = 72) | |
| --- | --- | --- | --- | --- | --- | --- | --- | --- |
|  | Freq. | % | Freq | % | Freq. | % | Freq | % |
| 0 | 52 | 24.6 | 13 | 14.4 | 15 | 30.6 | 24 | 33.3 |
| 1 | 7 | 3.3 | 1 | 1.1 | 1 | 2.0 | 5 | 6.9 |
| 2 | 5 | 2.4 | 0 | 0 | 2 | 4.1 | 3 | 4.2 |
| 3 | 7 | 3.3 | 3 | 3.3 | 1 | 2.0 | 3 | 4.2 |
| 4 | 9 | 4.3 | 5 | 5.6 | 0 | 0.0 | 4 | 5.6 |
| 5 | 13 | 6.2 | 3 | 3.3 | 6 | 12.2 | 4 | 5.6 |
| 6 | 12 | 5.7 | 4 | 4.4 | 2 | 4.1 | 6 | 8.3 |
| 7 | 9 | 4.3 | 3 | 3.3 | 3 | 6.1 | 3 | 4.2 |
| 8 | 7 | 3.3 | 4 | 4.4 | 0 | 0.0 | 3 | 4.2 |
| 9 | 11 | 5.2 | 2 | 2.2 | 5 | 10.2 | 4 | 5.6 |
| 10 | 5 | 2.4 | 5 | 5.6 | 0 | 0.0 | 0 | 0.0 |
| 11 | 13 | 6.2 | 5 | 5.6 | 7 | 14.3 | 1 | 1.4 |
| 12 | 9 | 4.3 | 6 | 6.7 | 1 | 2.0 | 2 | 2.8 |
| 13 | 6 | 2.8 | 3 | 3.3 | 1 | 2.0 | 2 | 2.8 |
| 14 | 6 | 2.8 | 5 | 5.6 | 0 | 0.0 | 1 | 1.4 |
| 15 | 5 | 2.4 | 3 | 3.3 | 0 | 0.0 | 2 | 2.8 |
| 16 | 3 | 1.4 | 2 | 2.2 | 1 | 2.0 | 0 | 0.0 |
| 17 | 5 | 2.4 | 3 | 3.3 | 1 | 2.0 | 1 | 1.4 |
| 18 | 5 | 2.4 | 4 | 4.4 | 0 | 0.0 | 1 | 1.4 |
| 19 | 4 | 1.9 | 3 | 3.3 | 0 | 0.0 | 1 | 1.4 |
| 20 | 3 | 1.4 | 1 | 1.1 | 1 | 2.0 | 1 | 1.4 |
| 21 | 6 | 2.8 | 4 | 4.4 | 1 | 2.0 | 1 | 1.4 |
| 22 | 5 | 2.4 | 4 | 4.4 | 1 | 2.0 | 0 | 0.0 |
| 23 | 1 | 0.5 | 1 | 1.1 | 0 | 0.0 | 0 | 0.0 |
| 24 | 3 | 1.4 | 3 | 3.3 | 0 | 0.0 | 0 | 0.0 |
|  | | | | | | | | |

**Table S13.** *Descriptive statistics of mark distribution for part C questions*

| Mark earned | All (*n* = 211) | | Instructor A  (*n* = 90) | | Instructor B  (*n* =49) | | Instructor C  (*n* = 72) | |
| --- | --- | --- | --- | --- | --- | --- | --- | --- |
|  | Freq. | % | Freq | % | Freq. | % | Freq | % |
| 2 | 1 | 0.5 | 0 | 0.0 | 0 | 0.0 | 1 | 1.4 |
| 4 | 3 | 1.4 | 0 | 0.0 | 1 | 2.0 | 2 | 2.8 |
| 5 | 3 | 1.4 | 2 | 2.2 | 0 | 0.0 | 1 | 1.4 |
| 6 | 3 | 1.4 | 2 | 2.2 | 1 | 2.0 | 0 | 0.0 |
| 7 | 2 | 1.0 | 0 | 0.0 | 1 | 2.0 | 1 | 1.4 |
| 8 | 6 | 2.8 | 3 | 3.3 | 2 | 4.1 | 1 | 1.4 |
| 9 | 4 | 1.9 | 0 | 0.0 | 1 | 2.0 | 3 | 4.2 |
| 10 | 3 | 1.4 | 0 | 0.0 | 1 | 2.0 | 2 | 2.8 |
| 11 | 1 | 0.5 | 0 | 0.0 | 0 | 0.0 | 1 | 1.4 |
| 12 | 4 | 1.9 | 2 | 2.2 | 0 | 0.0 | 2 | 2.8 |
| 13 | 5 | 2.4 | 0 | 0.0 | 2 | 4.1 | 3 | 4.2 |
| 14 | 3 | 1.4 | 2 | 2.2 | 0 | 0.0 | 1 | 1.4 |
| 15 | 11 | 5.2 | 3 | 3.3 | 2 | 4.1 | 6 | 8.3 |
| 16 | 13 | 6.2 | 2 | 2.2 | 5 | 10.2 | 6 | 8.3 |
| 17 | 7 | 3.3 | 2 | 2.2 | 1 | 2.0 | 4 | 5.6 |
| 18 | 10 | 4.7 | 2 | 2.2 | 4 | 8.2 | 4 | 5.6 |
| 19 | 8 | 3.8 | 4 | 4.4 | 0 | 0.0 | 4 | 5.6 |
| 20 | 9 | 4.3 | 4 | 4.4 | 1 | 2.0 | 4 | 5.6 |
| 21 | 15 | 7.1 | 5 | 5.6 | 7 | 14.3 | 3 | 4.2 |
| 22 | 6 | 2.8 | 4 | 4.4 | 0 | 0.0 | 2 | 2.8 |
| 23 | 13 | 6.2 | 6 | 6.7 | 4 | 8.2 | 3 | 4.2 |
| 24 | 11 | 5.2 | 5 | 5.6 | 4 | 8.2 | 2 | 2.8 |
| 25 | 12 | 5.7 | 6 | 6.7 | 2 | 4.1 | 4 | 5.6 |
| 26 | 7 | 3.3 | 5 | 5.6 | 2 | 4.1 | 0 | 0.0 |
| 27 | 7 | 3.3 | 4 | 4.4 | 2 | 4.1 | 1 | 1.4 |
| 28 | 5 | 2.4 | 2 | 2.2 | 1 | 2.0 | 2 | 2.8 |
| 29 | 4 | 1.9 | 1 | 1.1 | 0 | 0.0 | 3 | 4.2 |
| 30 | 3 | 1.4 | 1 | 1.1 | 0 | 0.00 | 2 | 2.8 |
| 31 | 5 | 2.4 | 3 | 3.3 | 1 | 2.0 | 1 | 1.4 |
| 32 | 4 | 1.9 | 1 | 1.1 | 2 | 4.1 | 1 | 1.4 |
| 33 | 5 | 2.4 | 4 | 4.4 | 0 | 0.0 | 1 | 1.4 |
| 34 | 6 | 2.8 | 4 | 4.4 | 1 | 2.0 | 1 | 1.4 |
| 36 | 4 | 1.9 | 3 | 3.3 | 1 | 2.0 | 0 | 0.0 |
| 37 | 6 | 2.8 | 6 | 6.7 | 0 | 0.0 | 0 | 0.0 |
| 38 | 1 | 0.5 | 1 | 1.1 | 0 | 0.0 | 0 | 0.0 |
| 39 | 1 | 0.5 | 1 | 1.1 | 0 | 0.0 | 0 | 0.0 |
|  | | | | | | | | |
